# Crosstalk between coagulation and complement activation promotes cardiac dysfunction in arrhythmogenic right ventricular cardiomyopathy

**DOI:** 10.7150/thno.58160

**Published:** 2021-04-03

**Authors:** Jie Ren, Konstantinos Tsilafakis, Liang Chen, Konstantinos Lekkos, Ioanna Kostavasili, Aimilia Varela, Dennis V. Cokkinos, Constantinos H. Davos, Xiaogang Sun, Jiangping Song, Manolis Mavroidis

**Affiliations:** 1 Department of Cardiac Surgery, State Key Laboratory of Cardiovascular Disease, Fuwai Hospital, National Center for Cardiovascular Diseases, Chinese Academy of Medical Sciences and Peking Union Medical College, China.; 2 Center of Basic Research, Biomedical Research Foundation, Academy of Athens, Athens, Greece.; 3 Clinical, Experimental Surgery & Translational Research Center, Biomedical Research Foundation, Academy of Athens, Athens, Greece.

**Keywords:** Arrhythmogenic right ventricular cardiomyopathy, complement, coagulation, proteomics, serum biomarkers.

## Abstract

**Aims:** We previously found that complement components are upregulated in the myocardium of patients with arrhythmogenic right ventricular cardiomyopathy (ARVC), and inhibiting the complement receptor C5aR reduces disease severity in desmin knockout (*Des^-/-^*) mice, a model for ARVC. Here, we examined the mechanism underlying complement activation in ARVC, revealing a potential new therapeutic target.

**Methods:** First, immunostaining, RT-PCR and western blot were used to detect the expression levels of complement and coagulation factors. Second, we knocked out the central complement component C3 in *Des^-/-^* mice (ARVC model) by crossing *Des^-/-^* mice with *C3^-/-^* mice to explore whether complement system activation occurs independently of the conventional pathway. Then, we evaluated whether a targeted intervention to coagulation system is effective to reduce myocardium injury. Finally, the plasma sC5b9 level was assessed to investigate the role in predicting adverse cardiac events in the ARVC cohort.

**Results:** The complement system is activated in the myocardium in ARVC. Autoantibodies against myocardial proteins provided a possible mechanism underlying. Moreover, we found increased levels of myocardial C5 and the serum C5a in *Des^-/-^C3^-/-^* mice compared to wild-type mice, indicating that C5 is activated independently from the conventional pathway, presumably via the coagulation system. Crosstalk between the complement and coagulation systems exacerbated the myocardial injury in ARVC mice, and this injury was reduced by using the thrombin inhibitor lepirudin. In addition, we found significantly elevated plasma levels of sC5b9 and thrombin in patients, and this increase was correlated with all-cause mortality.

**Conclusions:** These results suggest that crosstalk between the coagulation and complement systems plays a pathogenic role in cardiac dysfunction in ARVC. Thus, understanding this crosstalk may have important clinical implications with respect to diagnosing and treating ARVC.

## Introduction

Arrhythmogenic right ventricular cardiomyopathy (ARVC) is an inherited cardiomyopathy characterised by malignant arrhythmia and ventricular dysfunction, predominantly in the right ventricle 1-3; in some cases, the left ventricle can also be involved, particularly in progressive and end-stage disease 4. The pathological hallmarks of ARVC include progressive cardiomyocyte loss and fibrofatty replacement of myocardial tissue; in addition, infiltration of inflammatory cells can be observed in approximately 60-80% of patients 5. The general consensus is that the infiltration of inflammatory cells in the ventricular myocardium is associated with severe structural changes and is more prevalent in progressive cases with biventricular dysfunction 6-8. We previously reported that activation of the complement system plays a role in the progression of ARVC, as either knocking out or blocking the receptor for complement factor C5a (C5aR) significantly reduced myocardial remodelling 9 in a desmin knockout (*Des^-/-^*) mouse, a model for studying ARVC. Importantly, *Des^-/-^* mice recapitulate most of the pathognomonic features of ARVC 9.

In humans, mutations in the *DES* gene have been associated with severe human diseases, including various forms of myofibrillar myopathy and/or cardiomyopathy 10. Approximately 60% of patients have a cardiac conduction disease or arrhythmia, with atrioventricular block serving as an important clinical hallmark. In the past decade, an increasing number of reports describe patients who fulfil the so-called ARVC Task Force Criteria and are carriers of *DES* mutations 11-15. A recent report of the largest known family carrying a single *DES* mutation (DES-p.Glu401Asp), which predominantly causes inherited arrhythmogenic cardiomyopathy (ACM) 16, suggested that the prevalence of *DES* mutations in ACM is higher than previously described and is estimated to be 2-3% 17.

By performing deep-proteome analyses of explanted hearts from patients with end-stage ARVC, we previously found that several complement system components, including factors C3, C6, C7, C8, and C9, are significantly upregulated in both ventricles 18. Moreover, using weighted gene co-expression network analysis of patient samples, Chen et al. recently found that the *C5aR1* gene encoding complement receptor C5aR1 is one of four crucial hub genes in ARVC 19. These two studies support our previous findings in *Des^-/-^* mice, indicating that activation of the complement system may play an important role in the pathophysiology of ARVC via a currently unknown mechanism.

The complement system is a major component of innate immunity that not only acts to sense pathogens 20 but also by participating in a wide range of biological processes, including the clearance of immune complexes, angiogenesis, tissue regeneration, and lipid metabolism 21. However, insufficient, excessive, and poorly controlled complement activation can shift the balance from health to disease, thus contributing to a variety of immune-related and inflammatory diseases 22. The complement system is activated through three principal pathways known as the classical, alternative, and lectin pathways 20. All three pathways lead to the cleavage of C3 to C3a and C3b by the enzyme C3 convertase. In addition, C3 convertase can incorporate an additional C3b molecule, forming the enzyme C5 convertase, which cleaves C5. A novel form of complement activation via the coagulation cascade has also been described in C3-deficient mice, in which C5 is activated via thrombin-mediated proteolytic cleavage 23 (**Figure S1**). Since this original report back in 2006, a growing number of studies involving several models suggest that the thrombin-mediated generation of C5a is deleterious, including models of pulmonary contusion 24, tracheal transplant 25, arthritis 26, and transfusion of aged blood 27.

Recent studies have shown that circulating autoantibodies against cardiac and intercalated disc proteins are more prevalent among patients with ARVC compared to healthy controls and are associated with increased disease severity 28, 29. As described in several autoimmune diseases, an autoimmune complex ‒ primarily IgG ‒ can activate the complement system, resulting in a severe inflammatory response 30.

To investigate the detailed mechanism underlying the putative role of complement system activation in ARVC, we eliminated the key complement component C3 in the desmin knockout mouse model of ARVC by generating *Des^-/-^C3^-/-^* double-knockout mice, providing the first evidence that crosstalk between the complement and coagulation systems can exacerbate the underlying pathology in the context of cardiac injury. In addition, we found that activation of the complement system (measured as increased plasma sC5b9 levels) and activation of the coagulation system (measured as increased plasma thrombin levels) are correlated and are associated with poorer outcome in patients with ARVC. Thus, delineating the molecular links between the coagulation and complement systems may provide new therapeutic targets for ARVC, thereby improving clinical outcome.

## Methods

### Animal studies

Desmin knockout (*Des^-/-^*) mice were generated previously using standard methods 31 and are maintained on a 129SV and C57BL/6J mixed genetic background. C3 knockout (*C3^-/-^*) mice 32 were purchased from The Jackson Laboratory (Bar Harbor, ME) and are maintained on a C57BL/6 genetic background. To generate desmin and C3 double-knockout mice, *C3^-/-^* mice were backcrossed for 7 generations onto the *Des^-/-^* line. The mice were bred and housed under specific pathogen-free conditions at our institution's animal facility. Mice were euthanised by isoflurane inhalation followed by cervical dislocation. All procedures regarding the care and treatment of animals were approved by the Institutional Ethics Committee and the Animal Care Committee of East Attica County, Athens, Greece, and were performed in accordance with Directive 2010/63/EU of the European Parliament regarding the protection of animals used for scientific purposes.

### Human subjects

This study was approved by the Ethics Committee of Fuwai Hospital, Beijing, China, in accordance with standards established in the 1964 Declaration of Helsinki and its subsequent amendments. All participants provided written informed consent. All ARVC patients enrolled in the study were diagnosed using the 2010 revised Task Force Criteria 33, and patients with dilated cardiomyopathy (DCM) were diagnosed using the diagnostic criteria published by Mestroni et al. 34, for more details see the Online Supplementary Material methods.

### Statistical analysis

Continuous variables are expressed as the mean ± the standard error of mean (SEM) without special instructions, and categorical variables are presented as the number and percentage. For quantitative data, the Student's *t*-test or the Mann-Whitney *U* test was used for comparisons between two groups, and a one-way analysis of variance (ANOVA) was used to compare multiple groups. Categorical data were compared using the chi-square test or Fisher's exact test. *P*-values obtained from all multiple comparisons were adjusted using Tukey's or Sidak's method. The putative correlations between sC5b9 levels and clinical variables were examined using Spearman's test. The linear regression and Pearson's correlation test were used to determine the relationship between plasma sC5b9 and IgG/thrombin. Survival was analysed using Kaplan-Meier curves and compared using the log-rank test. Differences with a *P*-value (or adjusted *P*-value) <0.05 were considered statistically significant. All statistical analyses were performed using SPSS Statistics, version 23.0 (IBM Corp, Armonk, NY), and graphs were generated using GraphPad Prism 7 (GraphPad Software Inc., CA).

Detailed methods are provided in the Online Supplementary Material.

## Results

### The complement and coagulation systems are activated in the myocardium of patients with ARVC

**Figure 1:**
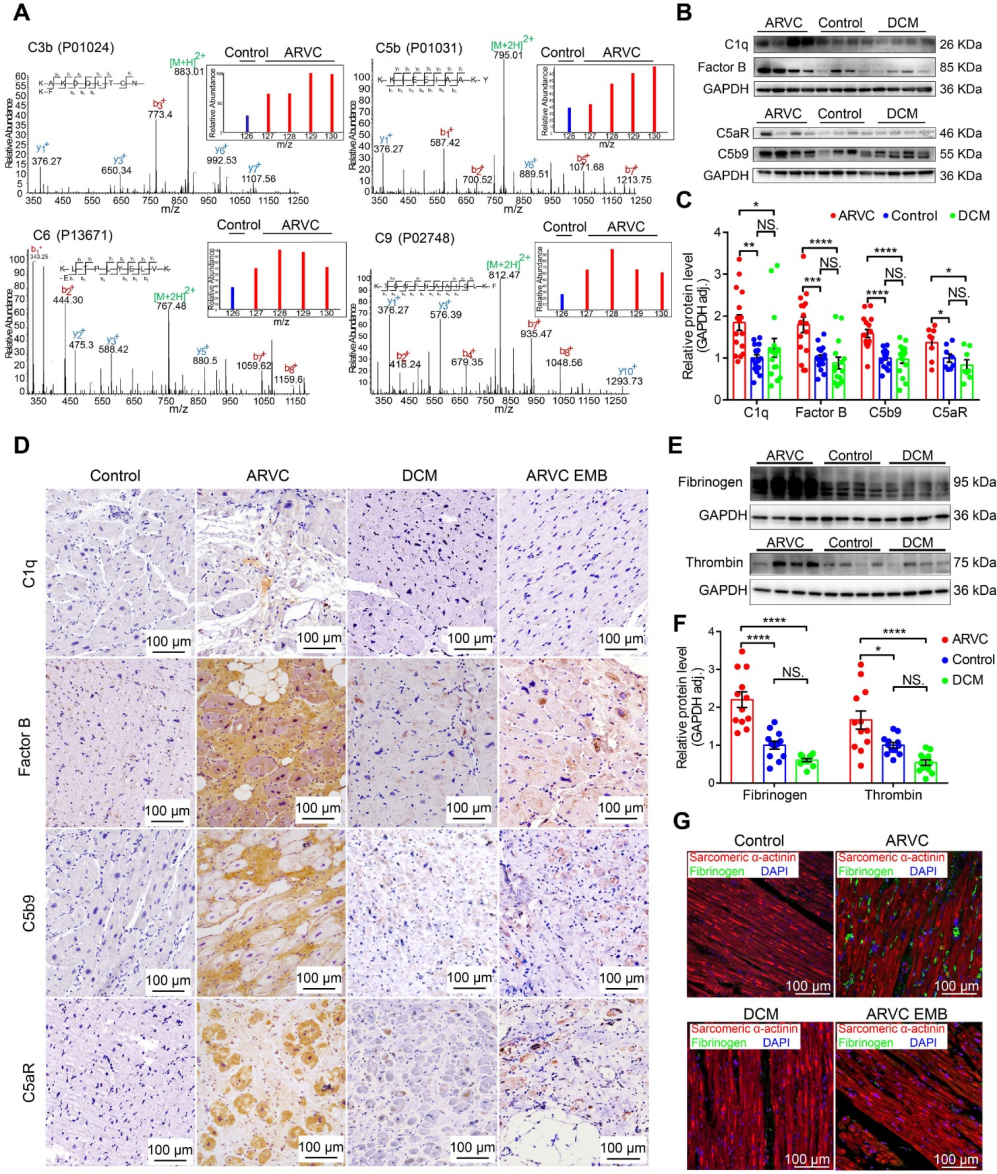
The complement and coagulation systems are activated in the myocardium of patients with end-stage ARVC. **A.** Proteomics analysis showing that most complement system factors were significantly increased in the right ventricle of ARVC patients. **B-C.** Representative western blot analysis and quantification of C1q, factor B, C5b9, and C5aR in the myocardium of healthy controls, ARVC patients, and DCM patients (n = 16/group); adjusted *P*-values were determined using a one-way ANOVA with Tukey's multiple comparison correction (number of comparisons=3). **D.** Immunohistochemical staining of C1q, factor B, C5aR, and C5b9 in cardiac tissue sections obtained from healthy controls, ARVC patients, DCM patients, endomyocardial biopsy (EMB) samples obtained from early-stage ARVC patients. **E-F.** Western blot analysis and quantification of fibrinogen and thrombin in the right ventricle of healthy controls, ARVC patients, and DCM patients (n = 12/group); adjusted *P*-value determined using a one-way ANOVA with Tukey's multiple comparison correction (number of comparisons=3).** G.** Immunofluorescence images of fibrinogen (green) and sarcomeric α-actinin (red) in cardiac tissue sections obtained from a healthy control, ARVC patient, DCM patient, and ARVC EMB; the nuclei were counterstained with DAPI. Shown are representative images (n = 4 subjects/group). * *P <* 0.05; ** *P <* 0.01, *** *P <* 0.001, **** *P <* 0.0001.

Myocardial tissue samples were obtained from controls (mixture of 4 non-diseased ventricles as the inner standards) and from patients with ARVC or DCM and used for quantitative proteomics analysis (**Table S1**). Secondary mass spectrography revealed that several complement system factors such as C3b and components of the C5b-C9 complex were significantly higher in the right ventricular myocardium of ARVC compared to control samples (**Figure 1A, Figure S2A**). Western blot analysis confirmed significantly higher levels of complement factors C5b9, factor B, and C1q in the myocardium of ARVC patients compared to control donors and DCM patients (**Figure 1B-C; Table S2:** Characteristics of ARVC patients in WB/qPCR; **Table S3:** Characteristics of DCM patients in WB/qPCR). In addition, expression of the pro-inflammatory receptor C5aR was also significantly higher in ARVC samples compared to control donors and DCM samples at both the protein (**Figure 1B-C**) and mRNA (**Figure S2B**) levels. These results were further supported by immunohistochemistry (IHC) showing that factor B, C5b9, and C5aR accumulated in the myocardial tissues in ARVC samples, particularly near regions of fibrofatty infiltration (**Figure 1D**). In contrast, virtually little complement protein deposits were observed in DCM or control myocardial tissues. (**Figure 1D**).

Western blot analysis confirmed significantly higher levels of fibrinogen and thrombin in ARVC samples compared to both control samples and DCM samples (**Figure 1E-F**). Using immunofluorescence, we confirmed that fibrinogen levels are higher in ARVC samples compared to both control and DCM samples (**Figure 1G**). Importantly, we also observed a modest increase of complement system proteins and coagulation factors (e.g. fibrinogen) in endomyocardial biopsy (EMB) samples taken from patients with ARVC in the early stages of the disease (**Figure 1D-G**).

Taken together, these results indicate that the complement and coagulation systems are robustly activated in right ventricular myocardium of ARVC but not in control or DCM.

### Autoimmune antibodies are associated with complement activation and deposition

**Figure 2:**
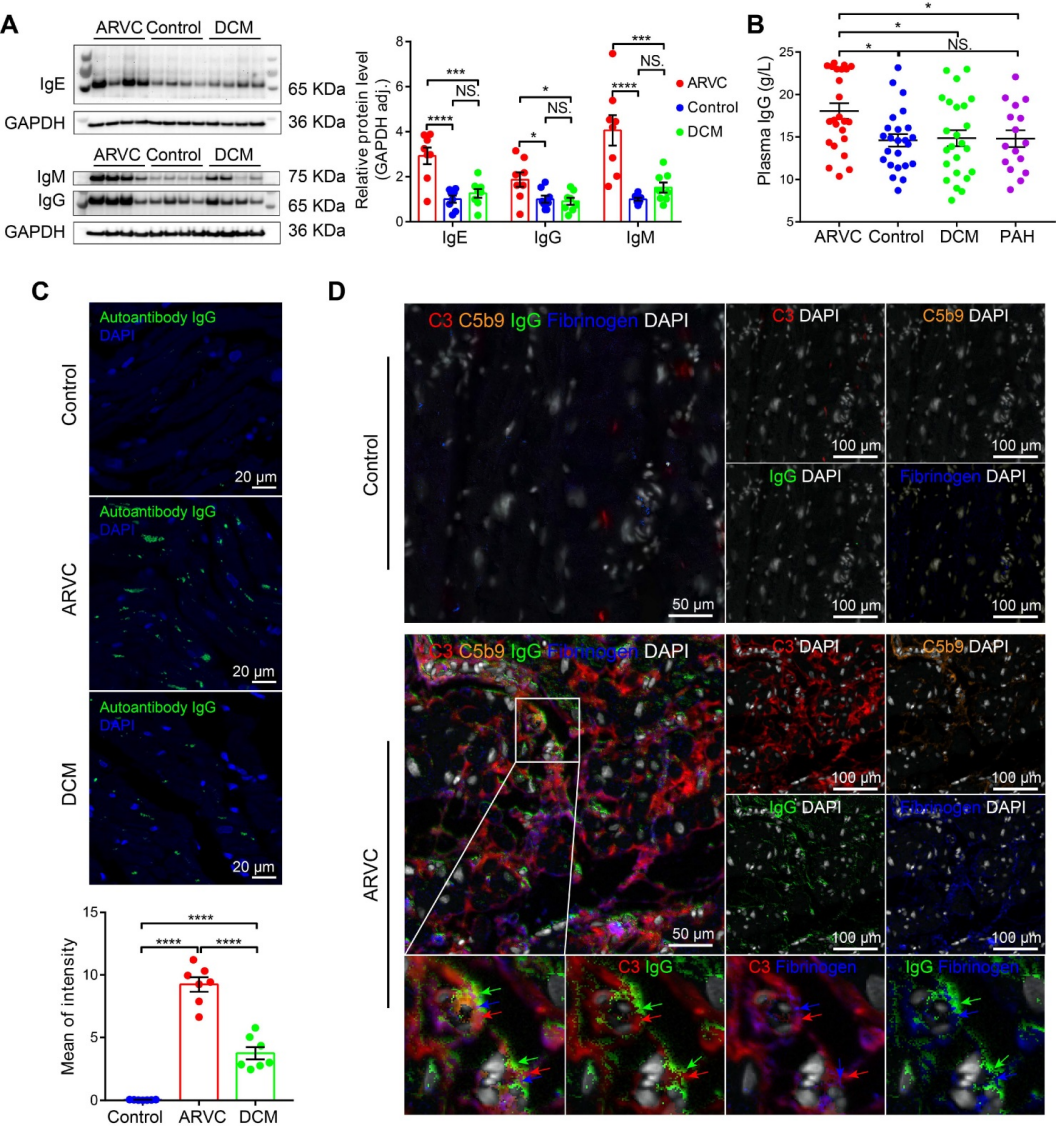
Autoimmune antibodies co-localise with complement proteins in the myocardium of ARVC patients. **A.** Western blot analysis and quantification of IgG, IgM, and IgE in the myocardium of healthy controls, ARVC patients, and DCM patients (n = 8/group); adjusted *P*-values were determined using a one-way ANOVA with Tukey's multiple comparison correction (number of comparisons=3). **B.** Summary of plasma IgG levels in healthy controls (n = 24), ARVC patients (n = 24), DCM patients (n = 24), and PAH patients (n = 16); adjusted *P*-values were determined using a one-way ANOVA with Tukey's multiple comparison test (number of comparisons=4).** C.** Autoantibody IgG staining in cardiomyocytes in a healthy control, ARVC patients, and DCM patient; the nuclei were counterstained with DAPI. **D.** Representative multiplex immunofluorescence images of cardiac sections obtained from a healthy control and an ARVC patient and immunostained for C3, C5b9, fibrinogen, and IgG. * *P <* 0.05; ** *P <* 0.01, *** *P <* 0.001, **** *P <* 0.0001.

Antibody-mediated activation of the complement system is an important component of the innate immune system, and recent studies showed that autoimmune antibodies are present in a relatively large proportion of families with ARVC 28, 29. We therefore examined the levels and distribution of autoimmune antibodies in the cardiac tissues of healthy controls, ARVC patients, and DCM patients. We found significantly higher levels of IgG, IgM, and IgE, in ARVC samples compared to both healthy controls and DCM samples (**Figure 2A**); in contrast, IgA and IgD were not significantly different between the three groups (**Figure S3A-B**). Moreover, IgG, the immunoglobulin that plays a critical role in complement activation, was also significantly higher in the plasma of ARVC patients compared to healthy controls, DCM patients, and pulmonary arterial hypertension (PAH) patients with right ventricular dysfunction (**Figure 2B**). Increased IgG deposits were also measured in both transplanted hearts and in EMB samples taken from early-stage ARVC patients (**Figure S3C**). Immunofluorescence also revealed higher levels of IgG autoantibodies against myocardial proteins in the plasma of ARVC patients compared to controls and DCM patients (**Figure 2C**). Using multiplex IHC, we also found distinct co-deposits of complement components C3 and C5b9, fibrinogen, and IgG in the myocardium of ARVC patients (**Figure 2D**). Immunofluorescence staining further confirmed the co-localisation of IgG and C3 in the myocardium of ARVC patients (**Figure S3D**).

We additionally analysed the sera of *Des^-/-^* mice for the presence of autoantibodies against cardiac tissue. IgG autoantibodies were identified against z-line proteins in 2 out of 12 *Des^-/-^* mice (**Figure S4**); a third mouse contained IgG autoantibodies against nuclear membrane proteins (data not shown). These results suggest that the deposition of autoimmune complexes ‒ primarily IgG ‒ may serve as the mechanism for activating the complement system in the myocardium of ARVC patients.

### Activation of the coagulation system exacerbates myocardial injury in ARVC mice

**Figure 3:**
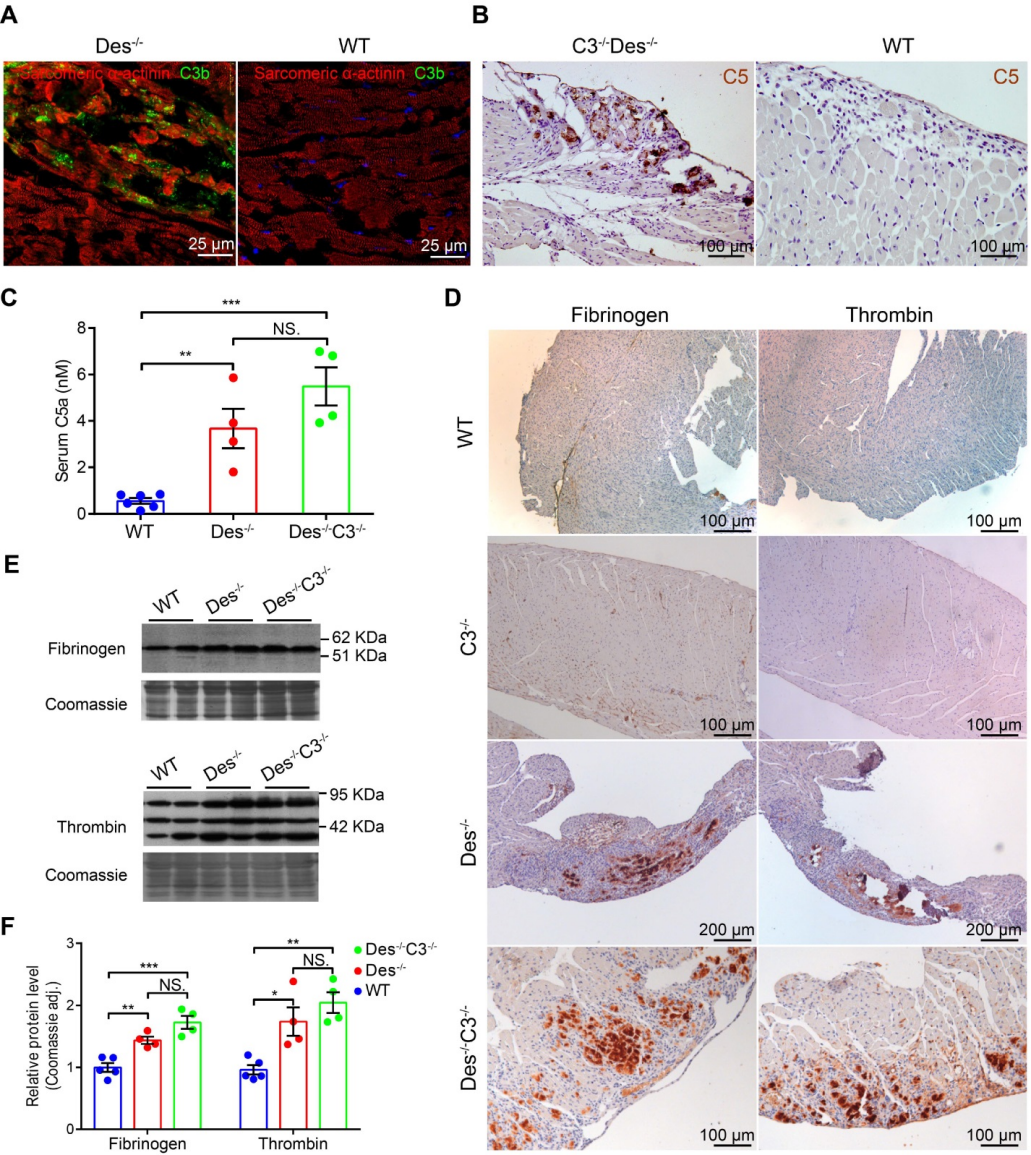
The complement and coagulation systems are activated in the myocardium of Des^-/-^ and C3^-/-^Des^-/-^ mice*.* **A.** Representative immunofluorescence images of frozen cardiac sections obtained from a *Des^-/-^* and WT mouse (B) immunostained with anti-C3b/iC3b (green) and anti- α-actinin (red). **C.** C5 immunohistochemical staining in the myocardium of a *Des^-/-^C3^-/-^* and WT mouse (**D**); shown are representative images (n = 8 mice/group). **E.** Summary of serum C5a concentration measured in WT, *Des^-/-^*, and *Des^-/-^C3^-/-^* mice; adjusted *P*-values were determined using a one-way ANOVA with Sidak's multiple comparison test (number of comparisons=3). **F.** Immunohistochemical staining of paraffin-fixed cardiac sections from the indicated mice stained for fibrinogen or thrombin. **G.** Western blot analysis and quantification **(H)** of fibrinogen and thrombin in the myocardium of WT, *C3^-/-^*, *Des^-/-^*, and *Des^-/-^C3^-/-^* mice; adjusted *P*-values were determined using a one-way ANOVA with Tukey's multiple comparison test (number of comparisons=3). * *P <* 0.05; ** *P <* 0.01, *** *P <* 0.001, **** *P <* 0.0001.

We previously reported massive complement activation in the myocardium of *Des^-/-^* mice 9. This transgenic model exhibit the initial degeneration that originates in the outer subepicardial myocardium in the right ventricle and follows a wave-live progression toward the endocardium 9; moreover, aneurysms often form in the so-called “triangle of dysplasia”, particularly following endurance training 1 (see **Figure S5**). In addition, *Des^-/-^* mice progressively develop heart failure 35 and a significant increase in premature ventricular contractions 9. Here, we found C3b deposits in areas containing necrotic cell debris and acute inflammatory infiltrates in *Des^-/-^* myocardium (**Figure 3A**) but not in the myocardium of wild-type (WT) mice (**Figure 3B**). A previous study suggested that coagulation factors ‒ particularly thrombin ‒ may promote activation of the complement system 23. In order to eliminate possible effects caused by activation of the classical, lectin, and alternative complement pathways (see **Figure S1**), we knocked out the central complement component C3 in *Des^-/-^* mice by crossing *Des^-/-^* mice with *C3^-/-^* mice, to produce *Des^-/-^C3^-/-^* double-knockout mice.

Given the loss of C3 protein in the *Des^-/-^C3^-/-^* mouse, we would have expected reduced cardiac tissue injury compared to the* Des^-/-^* single-knockout mouse due to impaired C5 activation. In contrast, we observed robust C5 staining in the myocardium of *Des^-/-^C3^-/-^*mice, particularly in areas with calcium deposits and tissue injury (**Figure 3C**), but not in WT myocardium (**Figure 3D**). Moreover, we measured significantly higher serum levels of C5a in both *Des^-/-^* and *Des^-/-^C3^-/-^*mice compared to WT mice (**Figure 3E**), indicating that C5 activation occurs independently of the conventional convertase pathway, possibly via activation of the coagulation system. Therefore, to determine whether crosstalk exists between the coagulation and complement systems in myocardium during ARVC, we stained cardiac tissue sections obtained from WT, *Des^-/-^*, and *Des^-/-^C3^-/-^* mice for components of the coagulation cascade. We observed robust immunostaining of fibrinogen and thrombin in the myocardium of *Des^-/-^* mice and *Des^-/-^C3^-/-^*mice (**Figure 3F**), particularly in areas of myocardial injury and dystrophic calcification, but not in WT, or *C3^-/-^*mice. We also observed increased immunostaining of von Willebrand factor in these areas in *Des^-/-^C3^-/-^* mice, (**Figure S6A**) but not in WT or* C3^-/-^* mice (**Figure S6B-C**). Consistent with the immunostaining results, western blot analysis confirmed increased levels of fibrinogen and thrombin in the myocardium of *Des^-/-^* and *Des^-/-^C3^-/-^*mice compared to WT mice (**Figure 3G-H**).

**Figure 4:**
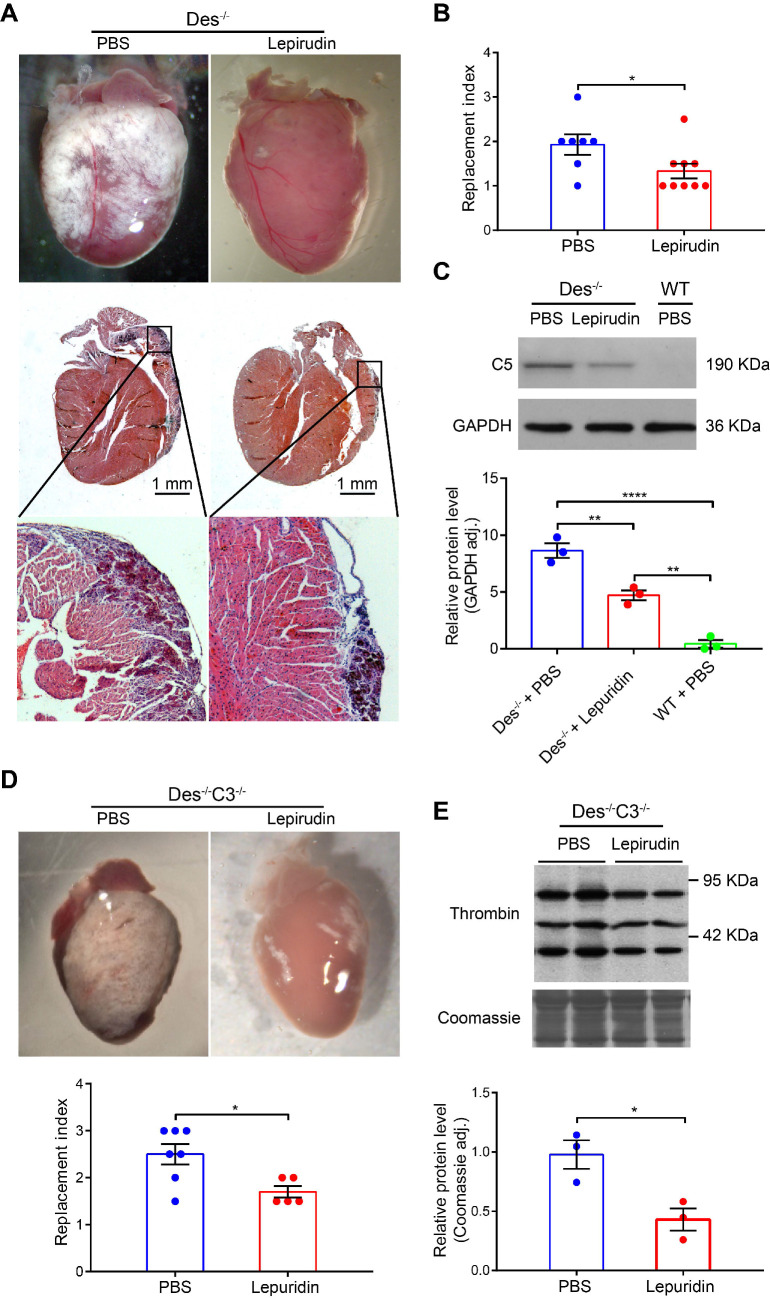
The thrombin inhibitor lepirudin reduces myocardial injury in Des^-/-^ and Des^-/-^C3^-/-^ mice. **A.** Whole-heart images as observed under the stereoscope and cardiac tissue sections (below) of the corresponding hearts (HE staining). **B.** Summary of replacement index in control (PBS) and lepirudin-treated *Des^-/-^* mice;* P*-value was determined using the Mann-Whitney *U* test. **C**. Western blot analysis and quantification of myocardial C5 in control-treated *Des^-/-^* mice, lepirudin-treated *Des^-/-^* mice, and WT mice; adjusted *P*-values were determined using a one-way ANOVA with Sidak's multiple comparison test (number of comparisons=3). **D.** Whole-heart images and summary of replacement index (below) in control (PBS) and lepirudin-treated *Des^-/-^ C3^-/-^* mice; *P*-value was determined using the Mann-Whitney *U* test. **E**. Western blot analysis and quantification of myocardial thrombin in control-treated and lepirudin-treated *Des^-/-^C3^-/-^* mice; *P*-value was determined using the Mann-Whitney *U* test. * *P <* 0.05; ** *P <* 0.01, *** *P <* 0.001, **** *P <* 0.0001.

Interestingly, we found that inhibiting activation of the coagulation system using lepirudin, a specific inhibitor of thrombin (see Materials and Methods) during the onset of myocardial injury (days 16-22) reduced fibrosis, calcification, and/or the infiltration of inflammatory cells (collectively referred to as the “replacement index”, see Supplementary Material and methods) by 25.5% compared to control-treated *Des^-/-^* mice (**Figure 4A-B**). Furthermore, the RNA levels of osteopontin and galectin 3 (markers of fibrosis, calcification 35), were also reduced (68.4-72.2%) in lepirudin-treated *Des^-/-^*animals compared to PBS-treated animals, as indicated by RT-PCR analysis of cardiac tissue extracts (**Table S4).** Lepirudin treatment also reduced the level of cardiac C5 protein in treated *Des^-/-^* mice (**Figure 4C**).

Similar results were obtained in *Des^-/-^C3^-/-^* mice with respect to reduced cardiac tissue pathology (“replacement index”, **Figure 4D**) and reduced thrombin protein levels (**Figure 4E**). These results suggest that activation of the coagulation system plays a causative role in the development of pathophysiology in *Des^-/-^* mice, possibly by activating the complement system.

**Figure 5:**
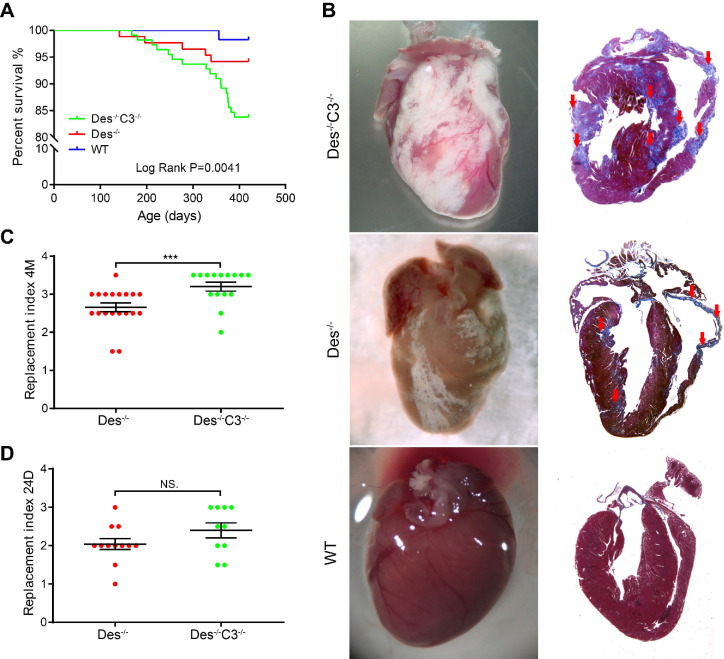
Des^-/-^C3^-/-^ mice have increased myocardial injury and reduced long-term survival. **A**. Kaplan-Meier survival curves for WT (n = 57), *Des^-/-^* (n = 86), and *Des^-/-^C3^-/-^* (n = 111) mice. The *Des^-/-^C3^-/-^* mice had significantly lower survival [83.8%, RR=9.96 (3.89-25.47), log-rank (Mantel-Cox) test, *P*=0.0041] compared to both WT (98.2%) and *Des^-/-^* (94.2%) mice; the survival rate was not significantly lower in the *Des^-/-^* group compared to WT (*P*=0.2330). **B**. Representative whole-heart images (left column) and Masson's trichrome‒stained sections (right column) from the indicated mice at 4 months of age. In the sections, blue staining indicates fibrosis (red arrows).** C**. Summary of replacement index in *Des^-/-^C3^-/-^* and *Des^-/-^* mice at 4 months (n = 10-15) and **(D)** 24-25 days of age (n = 12-17). *P*-values were determined using the Mann-Whitney *U* test. * *P <* 0.05; ** *P <* 0.01, *** *P <* 0.001, **** *P <* 0.0001.

With respect to survival, we found that *Des^-/-^C3^-/-^* mice have higher mortality compared to WT or *Des^-/-^* mice, during ageing, as 16.6% (13/78) of *Des^-/-^C3^-/-^*mice died spontaneously by 13 months, compared to only 4.3% (3/70) of the *Des^-/-^* mice (**Figure 5A**). At 12 months of age (when the increased mortality in *Des^-/-^C3^-/-^*mice is observed), echocardiography revealed a significant decrease in left ventricular fractional shortening in the* Des^-/-^C3^-/-^*mice compared to *Des^-/-^* mice, as well as increased left ventricular end-diastolic diameter and left ventricular end-systolic diameter (**Table S5**), indicating reduced cardiac function.

Interestingly, at 4 months of age we observed an 18% increase in cardiac tissue injury (estimated by grading the replacement index; see Materials and Methods) in Des*^-/-^C3^-/-^* mice compared to *Des^-/-^* mice (**Figure 5B-C**), despite no difference in left ventricular fractional shortening (**Table S6**). Histological analysis of cardiac tissue sections from *C3^-/-^* mice indicated a pattern similar to WT tissues (**Figure S6**). In contrast, at 24 days of age, we found no significant difference in cardiac tissue injury between *Des^-/-^C3^-/-^*mice and *Des^-/-^* mice (**Figure 5D**).

### Circulating complement levels are correlated with all-cause mortality events in ARVC patients

**Figure 6:**
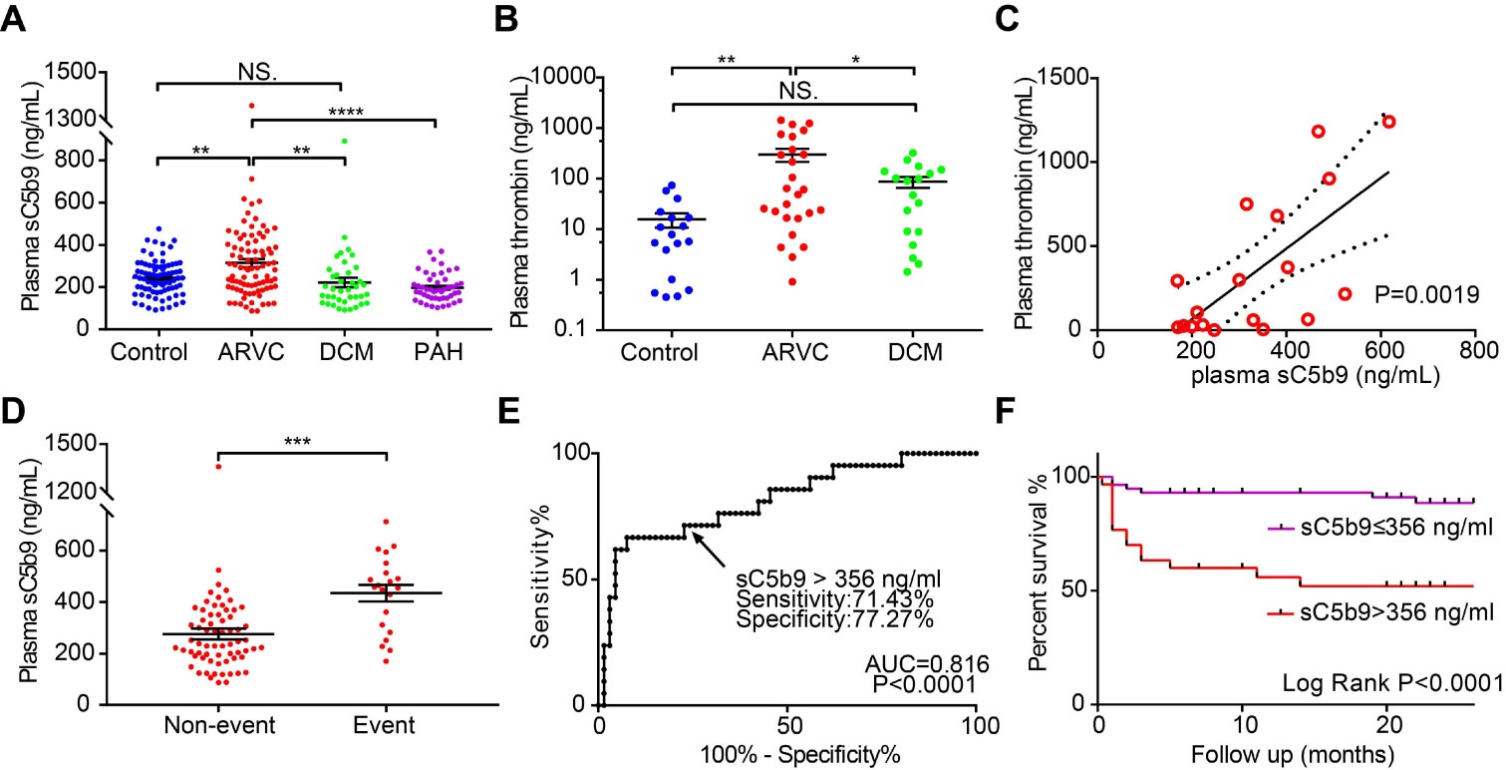
Circulating levels of complement factor sC5b9 are correlated with all-cause mortality events in ARVC patients. **A-B.** Summary of plasma sC5b9 levels and plasma thrombin levels in healthy controls, ARVC patients, DCM patients, and PAH patients with RV dysfunction; *P*-values were adjusted using Tukey's multiple comparison test (number of comparisons=3-4). **C.** Correlation between plasma thrombin concentration and plasma sC5b9 concentration in ARVC patients. The solid line represents the linear correlation, and the dotted lines represent 95% Confidence Intervals. **D.** Summary of plasma sC5b9 levels measured in ARVC patients without an event and ARVC patients with an all-cause mortality event during the follow-up period; the *P*-value was determined using the Student's *t*-test. **E.** Receiver operating characteristic (ROC) analysis used to determine the cut-off value for plasma sC5b9 concentration.** F.** Kaplan-Meier survival curves for ARVC patients with a plasma sC5b9 concentration >356 ng/mL and ARVC patients with a plasma sC5b9 concentration ≤356 ng/mL, showing significantly lower survival among the patients in the >356 ng/mL group (Log-rank (Mantel-Cox) test). * *P <* 0.05; ** *P <* 0.01, *** *P <* 0.001, **** *P <* 0.0001.

Lastly, we measured plasma sC5b9 levels in another cohort consisting of 79 healthy controls, 87 patients with ARVC, 39 patients with DCM, and 48 patients with PAH. The clinical data and serum complement factor levels are summarised in **Table 1** and are similar to previously reported cohorts 36, 37. We found that the plasma levels of sC5b9 were significantly higher in the patients with ARVC compared to the other three groups (**Figure 6A**). We then measured plasma sC5b9 levels in ARVC patients with different genotypes, including 17 patients with a mutation in *PKP2*, 12 patients with a mutation in *DSG2*, 3 patients with a mutation in *DSP*, 3 patients with a mutation in *DSC2*, and 1 patient with a mutation in *JUP*. Two out of the 87 ARVC patients (2.3%) had a mutation in the *DES* gene (DES p.R406W, DES p.R127C). Eight patients had more than one underlying mutation, with at least one desmosomal mutation **(Figure S7A**, detailed mutations shown in**Table S7)**. An ANOVA revealed that the plasma levels of sC5b9 did not differ significantly between the patient group with no mutation and the patient group with an underlying mutation regardless of whether we analysed all patients with mutations as a group (**Figure S7B**) or performed a subgroup analysis with desmosomal mutations, nondesmosomal mutations, and ≥2 mutations (**Figure S7C**). We also analysed the correlation between plasma sC5b9 levels and the clinical features in ARVC patients and found that elevated plasma sC5b9 levels were correlated with both ventricular dysfunction and arrhythmia severity, including major adverse cardiac events and atrial fibrillation (**Table S8**). Moreover, sC5b9 levels gradually but significantly increased with progressive cardiac involvement. We found that plasma sC5b9 levels in ARVC patients with biventricular dysfunction were significantly higher compared to healthy controls, patients without ventricular dysfunction, and patients with isolated right ventricular dysfunction (**Figure S8A**). We also found that plasma levels of the coagulation factor thrombin were higher in ARVC patients compared to both healthy controls and DCM patients (**Figure 6B**). Linear regression analysis revealed that plasma sC5b9 levels were positively correlated with plasma thrombin levels in ARVC patients (**Figure 6C**), suggesting crosstalk between the coagulation and complement systems in ARVC.

Finally, we examined the predictive value of plasma sC5b9 levels as a biomarker of all-cause mortality in ARVC patients. During a mean (± SD) follow-up period of 17.30 ± 9.51 months, a total of 21 patients reached the predefined endpoint, including receiving a heart transplant, death, or remaining on the heart transplant waiting list. Our analysis revealed that plasma sC5b9 levels among patients with an event were significantly higher compared to patients who did not experience an event during follow-up (**Figure 6D**). We then determined the optimum cut-off value for plasma sC5b9 concentration according to the likelihood ratio of receiver operating characteristic (ROC) diagnostic test (**Figure 6E**) and found that patients with a plasma sC5b9 concentration >356 ng/mL had a significantly higher rate of all-cause mortality compared to patients below this cut-off value (**Figure 6F**). We further compared the clinical and genetic differences of the ARVC patients with and without increased sC5b9 levels (>356 ng/mL) according to the ROC analysis (**Table S9**). Baseline characteristics and clinical data were roughly comparable between the groups, except for cardiac function (NYHA) and cardiac dilatation (RVID, LAAPD) There was no association between the levels of plasma sC5b9 and the gene mutation characteristics of ARVC patients These results are in accordance with above analyses of inter-group comparison (**Figure 6, Figure S6, S7**) and correlation analyses (**Table S8**). Although there were no significant group differences in the risk of arrhythmia, a significant increase in the occurrence of arrhythmias was observed in groups with elevated sC5b9 level (**Table S9**). These results suggested that sC5b9 levels gradually but significantly increased with progressive cardiac involvement. To further validate the relationship between plasma sC5b9 and adverse outcome in ARVC patients, we applied the Cox regression models to determine whether sC5b9 levels were associated with all-cause mortality in ARVC patients independently. We included the aforementioned variables, that were significantly associated with plasma sC5b9 level (**Table S8, S9**), and other well-described risk factors into the Cox model. Plasma sC5b9 level (HR=3.828, 95% CI= [1.031, 14.210] was identified as an independent prognostic factor for all-cause mortality in ARVC patients (**Table S10**).

These data indicate that elevated levels of plasma sC5b9 may serve as a potential predictor of adverse outcome in ARVC patients and support the hypothesis that complement components play a role in the pathophysiology of ARVC.

## Discussion

Here, we provide the first evidence of crosstalk between the coagulation and complement systems in the pathogenesis of ARVC, thereby exacerbating the underlying cardiac pathology. An association between the complement and coagulation systems has been suggested for a growing number of disorders 24-27 and was shown recently to have an adverse effect in patients with COVID-19 38. Interestingly, Huber-Lang et al. previously reported that C3-deficient mice can produce biologically active C5a due to the proteolytic activation of C5 by thrombin, independent of C5 convertases 23. Our results extend these original findings to the cardiac system by showing that C5 is activated in the myocardium and in the serum of *Des^-/-^C3^-/-^*mice in the apparent absence of the classical C5 convertase C3bC4b. Conversely, we observed increased activation of the coagulation system in the damaged myocardium of patients with ARVC, in *Des^-/-^*mice, and in *Des^-/-^C3^-/-^*mice. Moreover, our finding that inhibiting thrombin with lepirudin in both the *Des^-/-^* and *Des^-/-^C3^-/-^* mice reduces cardiac tissue injury supports the notion of crosstalk between the coagulation and complement systems and may have clinical relevance. For example, inhibiting the tissue factor-thrombin pathway has been shown to limit infarct size by reducing inflammation 39 and preventing cardiomyocyte cell death 40 in models of myocardial ischaemia-reperfusion injury. Interestingly, a relatively new anticoagulant, dabigatran, acts as a thrombin inhibitor and may therefore have clinically relevant implications with respect to treating patients with ARVC, as it can be administered both orally and long-term. Nomura Y et al found dissolution of a right ventricular thrombus with dabigatran, after heparin and anti-K anticoagulants had failed, indicating that direct thrombin inhibitors cam be active at the macro- and micro- level in patients with ARVC. Our results indicate that anticoagulant therapy could be administered in patients with ARVC in order to prevent the development of myocardial injury/remodelling and should not be reserved only for use in cases with atrial tachyarrhythmia and/or thromboembolic events. Atrial tachyarrhythmias ‒ which include atrial fibrillation and atrial flutter, two major causes of atrial thrombus formation ‒ are relatively common in patients with ARVC, developing in approximately 14-50% of patients 41. In our validation cohort of 87 patients with ARVC, 15 patients (17.2%) presented with atrial fibrillation; interestingly, plasma sC5b9 levels were significantly correlated with the presence of atrial fibrillation in patients with ARVC, but not in patients with DCM. We previously reported that the overall rate of right ventricular (RV) thrombus formation in ARVC patients to be 2.8% (13/467), while pulmonary embolism occurred in only one patient 42. Clinically, the patients with ARVC and RV thrombus presented with significantly impaired RV function and RV dilatation, and anticoagulation therapy led to complete thrombus resolution in 9 of these 13 patients (69%).

ARVC is considered a desmosomal-related disease, as mutations in desmosomal genes have been identified in ~50% of ARVC probands. Interestingly, however, mutations in a growing number of nondesmosomal genes have also been reported in patients with ARVC, including genes associated with other cardiomyopathies and arrhythmia syndromes such as desmin (*DES*), titin (*TTN*), lamin A/C (*LMNA*), Phospholamban (*PLN*), Transmembrane Protein 43 (*TMEM43*), and the sodium channel Nav1.5 (*SCN5A*) 17, 43. Moreover, non-genetic factors have been found to cause ARVC in approximately 30‒50% of cases 33, 34. Thus, the broader term arrhythmogenic cardiomyopathy (ACM) has been proposed to encompass the broader disease spectrum 44, including the classic form of ARVC 45, as well as the biventricular and left-dominant disease variants 46. ACM overlaps with other cardiomyopathies, particularly dilated cardiomyopathy with arrhythmia, which may be associated with ventricular dilatation and/or impaired systolic function 17.

The growing list of genes and clinical presentations involved in ACM indicates that the underlying mechanisms are still not fully understood and can include several components of the cellular “mechanochemical signalling and trafficking machinery”, which extend beyond the desmosome 44, 47. Desmin is believed to be an important element in this machinery, forming an extended scaffold that connects the entire contractile apparatus to the sarcolemma, the intercalated discs, the nucleus, mitochondria, lysosomes, and sarcoplasmic reticulum, and ‒ together with its associated proteins ‒ fine-tunes both mechanochemical signalling and trafficking, thus regulating the homeostasis and survival of cardiomyocytes 48, 49. Interestingly, all of the aforementioned proteins, for which mutations in their corresponding genes have been reported in patients with ACM, are localised in “structures” connected by the extended desmin scaffold 44, 48.

**Figure 7:**
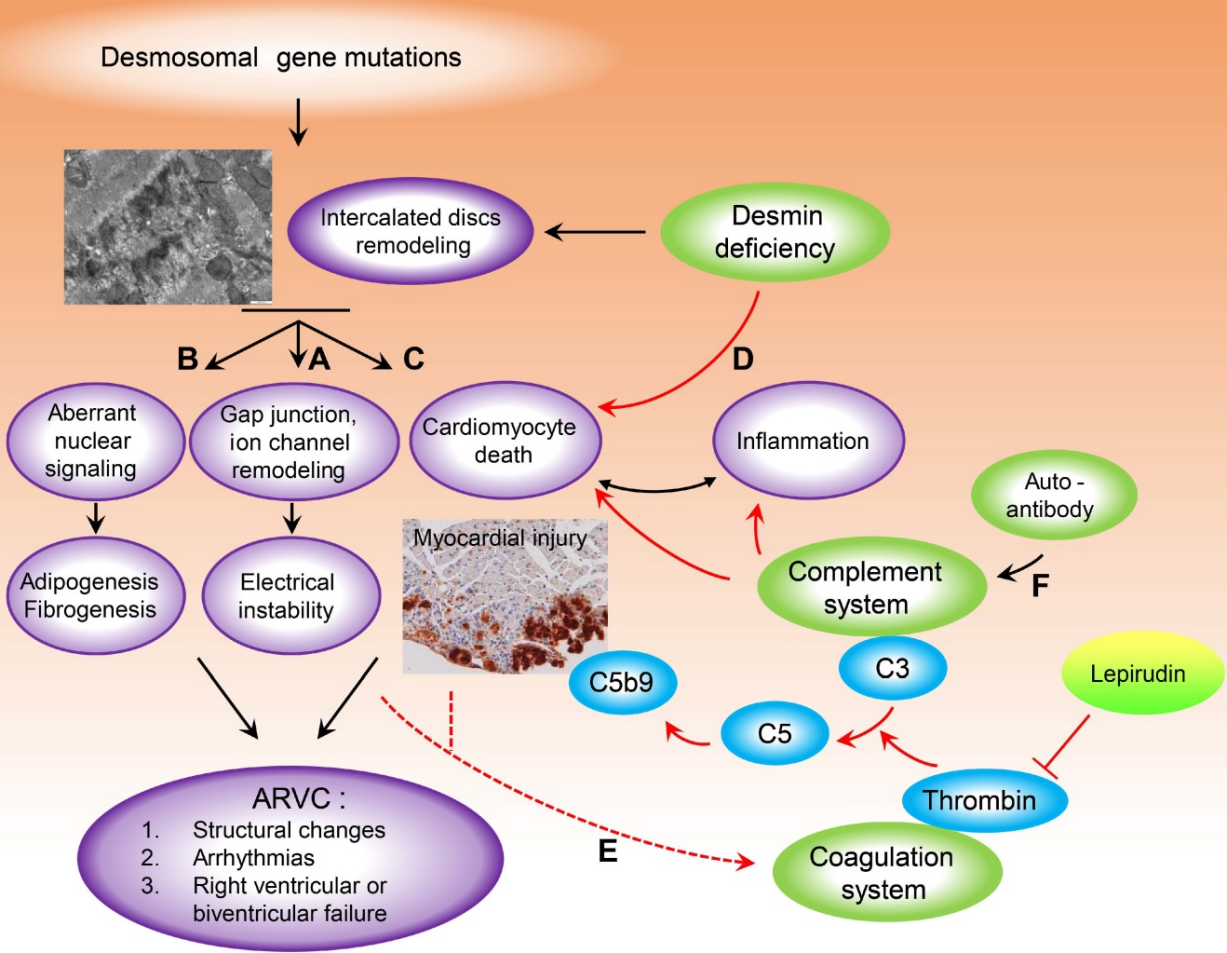
Model depicting the putative crosstalk between the complement and coagulation systems as a new pathogenetic mechanism that exacerbates myocardial injury in ARVC. Destruction of intercalated discs structural and functional integrity, due to desmosomal or desmin gene mutations are implicated in the pathobiology of arrhythmogenic cardiomyopathy. Abnormal biomechanical properties of desmosomes could affect the integrity of associated gap junctions and ion channels (**A**) and could explain the electrical instability, even in the absence of cardiac structural defects (concealed phase of the disease). (**B**) Aberrant nuclear signalling due to plakoglobin translocation from desmosomes to the nucleus, is thought to contribute to cardiomyocyte death and adipogenesis in ARVC. (**C**) The role of desmosomal gene mutations in the pathophysiology of myocardial injury characterised by sarcolemmal disintegration, myocyte death and the ensuing inflammatory reaction, observed in a lot of animal ARVC models, remains under investigation. As desmoplakin interacts directly with the muscle-specific intermediate filament desmin, cardiomyocyte death with sarcolemmal disintegration could also be the result of cytoskeletal impairment and loss of mechanochemical signalling (**D**). Cardiomyocyte injury and a systemic pro-coagulant state in ARVC patients due to bursts of arrhythmia could increase thrombus formation and complement activation at sites of injury, which could further enhance cardiac tissue destruction (**E**). Moreover, recent studies have suggested a higher prevalence of autoimmunity in ARVC probands and family members. We found significantly higher levels of IgG in the plasma and myocardium of ARVC patients, as well as co-localisation between IgG and complement proteins. IgG immune complexes can activate all three pathways of the complement system (**F**) which can drive severe inflammation. (For more details see Discussion). Red arrows indicate steps, pathways identified or proposed in this study. Black arrows indicate cellular and molecular cascades underlying arrhythmogenic cardiomyopathy proposed by previous studies, for review see 50, 67, 68.

Studies involving animal models and cellular models of ARVC have shown that abnormal biomechanical properties of the desmosome, which can affect the integrity and function of associated gap junctions and ion channels (**Figure 7A**) play a role in the pathobiology of ARVC and may explain the electrical instability even in the absence of structural cardiac defects (i.e. the “concealed” phase of the disease) 50. However, the role of mutations in desmosomal genes in the pathophysiology of myocardial injury characterised by sarcolemmal disintegration, myocyte necrosis, and the ensuing inflammatory reaction ‒ features that have been observed in many animal models of ARVC ‒ warrants further study 51 (**Figure 7B**). Dysregulation of the Wnt and Hippo pathways due to the translocation of plakoglobin from desmosomes to the nucleus is thought to contribute to the death of cardiomyocytes and adipogenesis in ARVC 52 (**Figure 7C**). Given that desmoplakin interacts directly with the muscle-specific intermediate filament desmin, cardiomyocyte death with sarcolemmal disintegration may also be the result of impaired cytoskeletal function and a loss of mechanochemical signalling 47, 48, 53 (**Figure 7D**). Indeed, a plethora of studies suggest structural and functional crosstalk between desmin and IDs, as mutations in desmin have been associated with changes in the structure of IDs 13, 54-56*.* Recently, Herrmann et al. found that R406W-desmin modifies the extra-sarcomeric cytoskeleton such that desmin filaments are not anchored to desmosomes, thereby destroying the structural and functional integrity of intercalated discs leading to myocardial injury 57. Here, we found that 2 of the 87 patients with ARVC (2.3%) in our study had a mutation in *DES*; one patient had the DES p.R406W, and the other patient had the DES p.R127C mutation.

Studies using animal models of ARVC have shown that loss of myocytes in either ventricle is an initiating event that subsequently triggers an inflammatory response and massive calcification within the myocardium, followed by injury repair with fibrous tissue replacement 51. Activation of the coagulation cascade is one of the earliest events initiated following tissue injury and requires a precisely coordinated temporal and spatial pattern in order to avoid bleeding and infection, control inflammation, and subsequently promote tissue repair. Moreover, dysregulation of the coagulation system can promote tissue degeneration via a wide range of mechanisms 58.

We observed increased activation of the coagulation system in the myocardium of patients with ARVC; in *Des^-/-^* mice, we observed increased levels of thrombin, fibrinogen, and von Willebrand factor, as well as deposits of activated C3, in areas of cardiac tissue injury. The C3a product of C3 activation has been implicated in regulating specific steps in thrombus formation, including platelet adhesion, Ca^2+^ spreading, and Ca^2+^ influx, by binding to the C3a receptor on platelets 59, potentially revealing an additional mechanism underlying the crosstalk between the complement and coagulation systems in the myocardium in ACM. In patients with ACM, a systemic pro-coagulant state due to bursts of arrhythmia such as atrial fibrillation and atrial flutter 60 may also increase thrombus formation and complement activation at the site of injury, thereby further increasing cardiac tissue damage (**Figure 7E**). An increased inflammatory state ‒ expressed clinically as increased levels of circulating C-reactive protein ‒ has been linked to ventricular tachycardia in patients with ARVC 61, and increased inflammation ‒ defined by increased serum levels of C-reactive protein, C3, and C4 ‒ has been found to increase the risk of atrial fibrillation 62. In addition, the complement system may also become activated as a nascent thrombus is forming, possibly playing a critical role in disease progression.

Recent studies have suggested a higher prevalence of autoimmunity in ARVC probands and their relatives 28, 29. Although this finding suggests that autoimmunity is involved in the pathogenesis and progression of ARVC, the underlying mechanism is currently unknown. In this respect, it is interesting to note that we found significantly higher levels of IgG in the plasma and myocardium of patients with ARVC, as well as co-localisation between IgG and complement proteins. IgG immune complexes can activate all three pathways of the complement system, thus driving severe inflammation 30. The infiltration of inflammatory cells and the resulting signalling activation is a pathological hallmark of ARVC 51, observed in 60-88% of patients 5, 6. Therefore, it is reasonable to speculate that the presence of autoimmune complexes in ARVC could serve as an additional mechanism contributing to the inflammatory response by activating the complement system (**Figure 7F**).

Knocking out C3 in desmin knockout mice led to increased cardiac pathology in the resulting double-knockout mice, with increased cardiac tissue injury and reduced survival in *Des^-/-^C3^-/-^* mice compared to mice lacking only desmin, and this may be attributed to activation of the coagulation cascade. Using an animal model of liver injury, He et al. found a C3 dose‒dependent balance between complement-dependent damage and regeneration 63, indicating that a threshold for C3 activation may determine whether it mediates tissue growth and regeneration or mediates pathophysiological processes 64, 65. Interestingly, using a model of acute lung inflammatory injury Huber-Lang et al. found higher plasma levels of thrombin activity in *C3^-/-^* mice compared to wild-type mice 23. Similarly, Khan et al. measured higher plasma levels of C5a in *C3^-/-^* mice compared to wild-type mice using a model of microvascular injury following allograft rejection 25; the authors speculated that a lack of opsonisation of the damaged microvasculature (due to the absence of C3) led to impaired clearance of cell debris, resulting in increased expression of tissue factor (the factor that initiates thrombin formation from prothrombin), activation of the coagulation cascade, and ‒ eventually ‒ increased C5 activation and injury. Interestingly, Acylation-Stimulating Protein (ASP), which has been identified as C3adesArg, is a product of C3 activation and participates in lipid and glucose utilizations, in different type of cells 66. Thus, the obligatory absence of C3adesArg from the *Des^-/-^C3^-/-^*mice could be an additional reason for increased cardiac pathology, as a result of miss-utilization of energy sources in this organism.

## Conclusions

Our results provide the first evidence of an increased prothrombotic state in the myocardium of ARVC patients and crosstalk between the complement and coagulation systems in an established mouse model of ARVC. Understanding this interplay between the complement and coagulation systems may have important clinical implications with respect to the diagnosis and prognosis of ARVC. Anticoagulant therapy with thrombin inhibitor could significantly reduce cardiac injury of ARVC mice, suggesting a promising translational perspective.

### Limitations

We recognise that our analysis may have been limited by the relatively small sample size; moreover, we were unable to adjust for all possible confounding variables related to the study's outcome. In this respect, additional multicentre studies involving large cohorts are needed in order to further investigate the putative value of determining prothrombotic state and complement activation in predicting clinical outcome in ARVC.

## Supplementary Material

Supplementary figures and tables.

## Figures and Tables

**Table 1:** Clinical characteristics of the cohort used to measure sC5b9 levels.

	Controls (N = 79)	ARVC (N = 87)	DCM (N = 39)	PAH (N = 48)
Male (%)	37 (46.84%)	58 (66.67%)	26 (66.67%)	28 (58.33%)
Age (years)	43.93±10.80	41.40±13.80	40.00±10.81	47.02±14.38
Weight (kg)	64.64±18.05	65.42±14.59	70.42±18.05	65.54±14.30
Height (cm)	165.27±7.21	168.31±11.31	169.52±7.21	168.00±9.13
BMI (kg/m^2^)	23.54±5.51	23.71±5.23	24.36±5.51	23.22±3.41
IVS (mm)	8.32±2.10	8.73±1.76	8.55±2.10	8.75±1.41
RVID (mm)	22.42±6.92	32.21±10.64	26.26±6.92	31.79±6.70
LVEDD (mm)	45.57±9.06	48.67±8.13	69.95±9.06	43.04±6.99
LVEF (%)	65.05±5.52	53.38±14.70	27.79±5.52	69.13±8.22
Plasma sC5b9 (ng/mL)	244.28±141.11	314.28±178.21	221.82±141.11	197.10±66.25

Data are expressed as either the mean ± SD or as the number (%). ARVC = arrhythmogenic right ventricular cardiomyopathy; DCM = dilated cardiomyopathy; PAH = pulmonary arterial hypertension; BMI = body mass index; IVS = interventricular septal thickness; RVID = right ventricular internal dimension; LVEDD = left ventricular end-diastolic diameter; LVEF = left ventricular ejection fraction.

## Abbreviations

ARVC: arrhythmogenic right ventricular cardiomyopathy
ACM: arrhythmogenic cardiomyopathy
SEM: standard error of mean
ANOVA: one-way analysis of variance
DCM: dilated cardiomyopathy
PAH: pulmonary arterial hypertension
IHC: immunohistochemistry
WT: wild-type
ID: intercalated discs
NYHA: New York Heart Association
RVID: Right ventricular internal dimension
LAAPD: Left atrium anteroposterior diameter
