# Supplementary material for: Crosstalk between coagulation and complement activation promotes cardiac dysfunction in arrhythmogenic right ventricular cardiomyopathy
Source: Theranostics. 2021 Apr 3;11(12):5939–54. doi: 10.7150/thno.58160 (PMC8058736; doi:10.7150/thno.58160)
Supplement: Supplementary file 1 — Supplementary figures and tables. [file thnov11p5939s1.pdf]

SUPPLEMENTARY MATERIAL

Supplementary methods

Animals studies

Desmin-null mice (*Des*$^{-/-}$) were previously generated by homologous recombination using standard methods[1] and are on a 129SV - C57BL/6 mixed genetic background. C3-null mice (*C3*$^{-/-}$) [2] were purchased from The Jackson Laboratory (Bar Harbor, ME) and are on a C57BL/6 genetic background. To generate desmin- and C3- double-deficient animals, *C3*$^{-/-}$ mice were backcrossed for 7 generations onto the *Des-/-* mice genetic background. Euthanasia of mice was achieved through inhalation of isoflurane (300μL in a 500ml container for drop jar anesthesia) followed by cervical dislocation. All mice were bred and housed under pathogen-free conditions at our institution's animal housing facility. The procedures for the care and treatment of animals were approved from the Institutional Ethical Committee and the Animal Care Committee of East Attica County, Athens, Greece, followed the guidelines from Directive 2010/63/EU of the European Parliament on the protection of animals used for scientific purposes.

Human subjects study design

This study was approved by the Ethics Committee of Fuwai Hospital, Beijing, China, in accordance with standards set out in the 1964 Declaration of Helsinki and its later amendments. All participants provided written informed consent. Firstly, we used 12-right ventricle (RV) specimens (4 ARVC, 4 DCM, 4 Normal) from the Heart Transplant Center of Fuwai Hospital for quantitative proteomics analysis (clinical baseline characteristics of heart transplantation patients are presented in **Table S1)**. Subsequently, in order to verify the results of proteomics, as well as for further Western blot, RT-qPCR and immunohistochemical analysis (see following paragraphs) we used myocardial tissues from additional 16 ARVC, 16 DCM and 16 normal specimens (see **Table S2** and **Table S3** for clinical characteristics of patients). Non-diseased hearts used for proteomics and histology analysis, were selected from those donor hearts initially considered and processed for transplantation but subsequently abandoned because of mismatched blood types or heart sizes. All of the non-diseased hearts had normal left ventricular function according to in

vivo evaluation and no history of myocardial disease. Endomyocardial biopsy specimens (EMB) derived from ARVC patients were also used to perform the above experiments.

Secondly, 87 ARVC patients, 39 DCM patients (both from the outpatients at early or intermediate stage), 48 pulmonary arterial hypertension (PAH) patients, and 79 healthy volunteers were enrolled to measure the circulating complement factor levels. Their clinical data are presented in **Table 1**. All the ARVC patients enrolled were diagnosed according to 2010 revised Task Force Criteria[3] and the DCM patients according to the diagnostic criteria of Mestroni et al.[4] To rule out the effects of genetic mutations, we have specially filtered out those DCM patients without gene mutation and family history (DCM patients used as a positive control of heart failure only). The clinical characteristics of the DCM cohort used to measure sC5b9 levels (N=39) are presented in Table 1. The characteristics of transplanted DCM patients (n=16) used in WB/qPCR analysis are presented in Supplementary Table S3. Baseline demographics and medical details of patients, including symptoms, NYHA class, comorbidities, arrhythmia occurrence, noninvasive and invasive examination (ECG, Echocardiography, CMR, and biochemical tests) were obtained retrospectively from the chart review by persons blinded to the assay results. Age of onset was defined as the age when the first symptoms or signs most likely to be attributable to the disease occurred. Detailed family history was acquired by ARVC genetic counseling, as well as comprehensive mutation testing. The patient questionnaires or telephone interview regarding information about the adverse cardiovascular events, including the all-cause death, heart transplantation or in waiting list, ventricular fibrillation (VF) and ICD discharge, were updated regularly after patient enrollment.

Genetic screening

The whole-genome DNA was extracted from peripheral blood cells of ARVC patients. Targeted next-generation sequencing was performed based on the Hi-seq2000 platform (Illumina, USA). The ARVC related genes such as *PKP2*, *DSG2*, *DSC2*, *JUP*, *DSP*, *TMEM43*, *LMNA*, *DES*, *SCN5A*, *CDH2* etc were screened. The pathogenicity of the variants was filtered and evaluated by ACMG guidelines, as previously described[5].

Proteomic analysis

Right ventricle (RV) specimens from 4 ARVC patients, 4 DCM patients, 4 non-diseased donors were selected for quantitative proteomic analysis. The donor hearts were selected from those abandoned because of mismatched blood types or heart sizes. The clinical baseline characteristics of HTx ARVC and DMC patients involved in proteomics analysis are presented in supplementary Table 1. To quantitate the proteins in each sample, TMT reagent (Thermo, Pierce Biotechnology, Rockford, USA) was applied to each sample. Quantitative proteomics analysis based on liquid chromatography−tandem mass spectrometry (LC−MS/MS) was performed as previously described.[6]

Western Blot

The procedures were conducted as previously reported in RV cardiac tissue extracts, except other way indicated. The antibodies included: Factor B (ab176024, abcam, UK), C1q (ab71940, abcam, UK), C5b9 (ab55811, abcam, UK), C5aR (ab59390, abcam, UK), Anti C5 (goat sc21938, Santa Cruz Germany), Thrombin (ab83981, abcam, UK), fibrinogen (ab34269, abcam, UK), IgA (ab193138, abcam, UK), IgD (ab124795, abcam, UK), IgE (ab99806, abcam, UK), IgG (ab200699, abcam, UK), IgM (ab212201, abcam, UK), and GAPDH (ab8245, abcam, UK). GAPDH was used as the internal reference of the protein expression. The clinical baseline characteristics of HTx ARVC and DMC patients involved in WB analysis are presented in supplementary Tables 2 and 3.

RT-qPCR

The relative gene expression levels in human cardiac samples (RV) were detected by a SYBR green based PCR kit (Applied Biosystems, Foster City, CA, USA). The specific protocols for RNA extraction and reverse transcription were described in a previous study. The ribosomal protein L5 (*RPL5*) was used as the internal reference gene of the qRT-PCR. The ΔΔCt method was used for the calculation of relative gene expression levels. The primer pairs for qRT-PCR were; *C5aR*: forward 5'- TATCCACAGGGGTGTTGAGG -3' and reverse 5'-GCCCAGGAGACCAGAACAT -3'.

The relative gene expression levels in mouse cardiac samples were performed in a similar way, as for humans, and as previously described[10.] Primer pairs targeting sequential exons of individual genes were as follows: osteopontin (forward: 5'TGATTCTGGCAGCTCAGAGG3';

reverse: 5’CTGTGGCGCAAGGAGATTCT3’; galectin-3 (forward: 5’GTGCCCTATGACCTGCCCTT3’; reverse: 5’GGCAACATCATTCCCTCTCC3’. Normalization for the amount of template was done using primers specific for the mouse β-actin gene (forward: 5’TGGCTCCTAGCACCATGA3’; reverse: 5’CCACCGATCCACACAGAG3’.

Immunofluorescent/immunohistochemical staining

Mice cardiac tissue sections (10μm thick) were fixed with acetone/methanol 1:1 at -20°C for 20 minutes and then used for immunofluorescence/immunohistochemical analysis. The samples were stained using the following primary antibodies: anti-C3b/iC3b/iC3c monoclonal antibody (clone 2/11, 1:80, HyCult Biotechnology, Netherlands), anti-sarcomeric α-actinin monoclonal antibody (clone EA-53, 1:100, Sigma, St Louis, Missouri, USA), anti-C5 (sc21938, goat, 1:100), anti-fibrinogen (ab34269, rabbit, 1:700, Abcam, Cambridge, UK), anti-von Willebrand factor (vWF) (#0082, rabbit, 1:200, DakoCytomation, Denmark), anti-C3 (ab97462, rabbit, 1:400, Abcam, Cambridge, UK) and anti-IgG (ab200699, mouse, 1:100, Abcam, Cambridge, UK). For confocal imaging, a Leica TCS SP5, DMI6000, microscope (inverted, with the acquisition software LAS-AF, at 23–24°C; Leica Microsystems, Wetzlar, Germany) was used. For the immunohistochemistry (IHC) assay of human cardiac samples, the paraffin sections of myocardial tissue were incubated with a primary antibody in a moist chamber overnight at 4℃. Primary antibodies included anti-factor B (ab176024, 1:100, abcam, UK), anti-C1q (SAB4502986, 1:50, sigma, USA), anti-C5b9 (ab55811, 1:1000, abcam, UK) and anti-C5aR (ab59390, 1:500, abcam, UK). The secondary antibody was incubated for 1 hour at room temperature, and the antibody-peroxidase complex was developed using DAB chromogen at room temperature for 2–5 min. The immunohistochemical images were scanned by the AxioScan.Z1 slide scanner (Zeiss, Germany)

Autoantibody detection

For the detection of autoantibody in plasma, indirect immunofluorescence (IIF) was performed on the unfixed fresh-frozen sections of normal human ventricular tissue as previously reported[7], using a 1/10 dilution of the plasmas from healthy controls and patients with ARVC and DCM. The immunofluorescence preparations were analysed through confocal microscopy (Zeiss LSM710/780; Zeiss, Germany).

In order to evaluate the presence of autoantibodies in mice sera, immunofluorescent analysis of 12μm thick heart sections was performed eventually as described in the previous paragraph (Immunofluorescent/immunohistochemical staining). The antibodies included: Des-/- and wt mouse sera in a 1:100 dilution and anti-desmin (H76, 1:50, Santa Cruz Biotechnology, Dallas, Texas, USA) and anti-N-cadherin (Alomone labs, Israel 1:100).

Multiplex immunohistochemistry

In order to detect the co-expression of multiple proteins such as complement, coagulation and IgG in the myocardium, using a single section, a multiplex immunohistochemistry array was conducted using the Opal Polaris 7-Color Automation IHC Kit (Akoya Biosciences, Marlborough, MA, USA; NEL871001KT) according to the manufacturer's instructions. Antibody panels were C3(ab97462, abcam, UK), C5b9 (ab55811, abcam, UK), fibrinogen (ab34269, abcam, UK) and IgG (ab200699, abcam, UK). The Opal fluorophores (Opal 520, Opal 540, Opal 570, Opal 620) were used at a 1 in 150 dilution, as recommended (Akoya Biosciences, USA). The acquired images (fluorescently labelled) were scanned on the Vectra Polaris™ at 20x magnification (PerkinElmer, USA), and multispectral unmixing was performed using inForm 2.0 (PerkinElmer, USA).

Assessment of replacement index

Routine histologic procedures and staining with hematoxylin-eosin (H-E) and Masson's trichrome were performed in paraffin-fixed sections. To assess the "replacement index," which represents the areas of cardiac tissue replaced by fibrosis and/or calcification and/or infiltration of inflammatory cells, the hearts were sectioned across the longitudinal axis (at the midline), and eight sections per heart (including the right and left ventricular free walls and the septum as indicated in Figure 5B) were analyzed by two independent, blinded observers as previously described[8]. Sections were graded from 0 to 4 as follows: 0= no tissue injury, 1=one or two foci of limited replacement, usually in the right ventricle; 2=two to three foci in two different areas of the cardiac tissue; 3=multiple foci of extended replacement, usually in all ventricular compartments (right and left ventricular free walls and the septum); 4=multiple foci of extended and diffuse fibrosis occupying more than 50% of the myocardial surface in a given section. A

Leica DMRA2 microscope was used for all bright field microscopy, and photographs were taken using a Leica DFC 500 camera and the Leica Application suite V3.6 program (Leica Microsystems, Wetzlar, Germany).

Treatment of young mice with the thrombin inhibitor lepirudin.

Des$^{-/-}$ or *Des*$^{-/-}$ *C3*$^{-/-}$ mice were treated with injections into the peritoneal cavity of lepirudin (Refludan, Hoechst Marion Roussel, Athens Greece), a highly specific direct inhibitor of thrombin. At day 16 after birth, before the onset of the acute inflammatory reaction, Des$^{-/-}$ animals were injected with 20mg (per kg body weight) of lepirudin, every 12 hrs for 5 consecutive days. Control *Des*$^{-/-}$ animals were injected with PBS. After the treatment, the animals were sacrificed and cardiac tissue sections were analyzed for infiltration of inflammatory cells and/or fibrosis and/or calcification as described above (replacement index).

Echocardiography

Echocardiographic experiments were performed in male mice of all genetic permutations at the age of 4 and 12 months, using an ultrasound system (Vivid 7; GE Healthcare, USA) with a 13-MHz linear transducer, as previously described[8]. Mice were anesthetized with an intraperitoneal injection of 50 to 100 mg/kg ketamine.

Measurements of circulating complement factors

Enzyme-linked immunosorbent assays (ELISA) was used to measure circulating levels of C1q (BMS2099, Thermo, USA), Factor B (A027, Quidel, USA), sC5b9 (A029, Quidel, US), and thrombin (ab108907, abcam, UK) in human plasma; all tests were performed according to the manufacturers' protocols.

Mouse serum was analyzed for C5a/C5adesArg concentrations, by the Biacore 2000 instrument (GE Healthcare, Munich, Germany) using the anti-C5a antibody (R&D Systems, clone 295108, MAB21501) as previously described[8].

References

1. Milner DJ, Weitzer G, Tran D, Bradley A, Capetanaki Y. Disruption of muscle architecture and myocardial degeneration in mice lacking desmin. J Cell Biol. 1996; 134: 1255-70.
2. Wessels MR, Butko P, Ma M, Warren HB, Lage AL, Carroll MC. Studies of group B streptococcal infection in mice deficient in complement component C3 or C4 demonstrate an essential role for complement in both innate and acquired immunity. Proc Natl Acad Sci U S A. 1995; 92: 11490-4.
3. Marcus FI, McKenna WJ, Sherrill D, Basso C, Bauce B, Bluemke DA, et al. Diagnosis of arrhythmogenic right ventricular cardiomyopathy/dysplasia: proposed modification of the Task Force Criteria. Eur Heart J. 2010; 31: 806-14.
4. Mestroni L, Maisch B, McKenna WJ, Schwartz K, Charron P, Rocco C, et al. Guidelines for the study of familial dilated cardiomyopathies. Collaborative Research Group of the European Human and Capital Mobility Project on Familial Dilated Cardiomyopathy. Eur Heart J. 1999; 20: 93-102.
5. Chen L, Rao M, Chen X, Chen K, Ren J, Zhang N, et al. A founder homozygous DSG2 variant in East Asia results in ARVC with full penetrance and heart failure phenotype. Int J Cardiol. 2019; 274: 263-70.
6. Chen L, Yang F, Chen X, Rao M, Zhang NN, Chen K, et al. Comprehensive Myocardial Proteogenomics Profiling Reveals C/EBPalpha as the Key Factor in the Lipid Storage of ARVC. J Proteome Res. 2017; 16: 2863-76.
7. Caforio ALP, Re F, Avella A, Marcolongo R, Baratta P, Seguso M, et al. Evidence From Family Studies for Autoimmunity in Arrhythmogenic Right Ventricular Cardiomyopathy: Associations of Circulating Anti-Heart and Anti-Intercalated Disk Autoantibodies With Disease Severity and Family History. Circulation. 2020; 141: 1238-48.
8. Mavroidis M, Davos CH, Psarras S, Varela A, N CA, Katsimpoulas M, et al. Complement system modulation as a target for treatment of arrhythmogenic cardiomyopathy. Basic Res Cardiol. 2015; 110: 27.

Table S1. Clinical baseline of individuals involved in proteomics analysis

Individual	Gender	BMI	Age at onset	Age at HTx	Age	LVEF	NYHA
ARVC-1	Male	26.4	20	22		60	IV
ARVC-2	Female	22.35	40	40		38.7	III
ARVC-3	Male	28.1	27	30		50.4	IV
ARVC-4	Male	20.8	27	34		41	IV
DCM-1	Male	22.7	36	38		25	IV
DCM-2	Female	24.3	44	47		25	IV
DCM-3	Male	19.6	18	21		19	III
DCM-4	Female	20.8	35	39		31	IV
Control-1	Female	23.3			21	66.9	
Control-2	Male	25.7			35	60.2	
Control-3	Male	24.2			53	62.7	
Control-4	Male	23.8			46	65.8	

ARVC = arrhythmogenic right ventricular cardiomyopathy; DCM = dilated cardiomyopathy; BMI= body mass index; HTx = heart transplantation; LVEF = left ventricular ejection fraction; NYHA= New York Heart Association;

Table S2. Characteristics of ARVC patients in WB/qPCR

Patient no. *	Sex	Age at HTx	BMI (kg/m^2)	Global or regional dysfunction and structural alterations	Tissue characterization of wall	Repolarization abnormalities	Depolarization/conduction abnormalities	Arrhythmias	Family history	Sum
				Major/Minor	Major/Minor	Major/Minor	Major/Minor	Major/Minor	Major/Minor	Major/Minor
1	M	37	20.9	0/1	1/0	1/0	1/0	1/0	0	4/1
2	F	41	21.3	1/0	1/0	NA	0	0	1/0	3/0
3	F	27	15.8	1/0	1/0	0	1/0	0	0/1	3/1
4	M	61	20.4	1/0	1/0	0	0	1/0	0/1	3/1
5	M	43	25.2	0/1	1/0	0/1	0	0	0	1/2
6	M	43	20.4	1/0	1/0	1/0	0	0	0/1	3/1
7	M	37	18.8	1/0	0/1	0/1	0	1/0	0	2/2
8	M	16	22.5	1/0	1/0	1/0	1/0	0	0	4/0
9	M	48	19	1/0	1/0	0/1	0	1/0	0/1	3/2
10	F	31	16.8	1/0	1/0	0	1/0	0/1	0	3/1
11	M	14	16	1/0	1/0	0/1	1/0	0	1/0	4/1
12	M	23	19	1/0	1/0	NA	NA	0/1	0	2/1
13	M	36	28.4	1/0	1/0	0	1/0	1/0	1/0	5/0
14	M	32	24.2	1/0	1/0	0/1	1/0	0	1/0	4/1
15	M	23	28.4	1/0	1/0	0/1	1/0	1/0	0	4/1
16	F	63	22.1	1/0	1/0	0/1	0	0/1	1/0	3/2

ARVC = arrhythmogenic right ventricular cardiomyopathy; BMI = body mass index; HTx = heart transplantation; NA, data unavailable; M = male; F = female

Table S3. Characteristics of DCM patients in WB/qPCR

Patient NO.	Sex	Age at HTx	BMI (kg/m^2)	LVEF (%)	LVEDD (mm)	Family history
1	M	40	19.8	28	64	no
2	M	35	24.3	25	78	no
3	F	49	20.8	34	67	no
4	M	28	21	18	78	no
5	M	39	24.7	24	24	no
6	M	31	22.1	17	62	no
7	M	36	20.6	24.8	80	no
8	M	59	20.1	25	75	no
9	F	38	22.7	25	66	no
10	F	27	19.2	30	62	no
11	M	25	21.8	25	63	no
12	F	37	18.7	28	80	no
13	F	44	20.3	18.3	65	no
14	M	39	22	20	79	no
15	F	26	19.4	19	77	no
16	M	43	18.2	23	89	no

DCM = dilated cardiomyopathy; BMI = body mass index; HTx = heart transplantation; LVEDD = left ventricular end diastolic dimension; LVEF = left ventricular ejection fraction. M = male; F = female

Table S4. Gene expression analysis by real-time PCR, in Des$^{-/-}$ mice treated with Lepirudin.

	**Des$^{-/-}$/PBS (fold increase vs. WT)**	**Des$^{-/-}$ Lepirudin (fold increase vs. WT)**	**% reduction (Des$^{-/-}$/PBS vs. Des$^{-/-}$ Lepirudin**
	(n=4)	(n=4)	
Osteopontin	62.7±7.6	17.4±2.5*	72.2%
Galectin 3	22.8±4.1	7.2±1.6†	68.4%

Real-time PCR data showing fold increase in expression of osteopontin and galectin-3 in Des$^{-/-}$/PBS and Des-/- Lepirudin treated hearts when compared to WT controls (n=4).

* p=0.0013 versus Des$^{-/-}$/PBS

†p=0.028 versus Des$^{-/-}$/PBS

Mann-Whitney test

Table S5. Echocardiography analysis of 12 months old mice

**Table 1**	**Des$^{-/-}$C3$^{-/-}$**	**Des$^{-/-}$**	**Wildtype**
	**n=7**	**n=9**	**n=8**
Heart Rate	490.47±13.39*	541.07±13.21	581.66±25.63
LVEDD(mm)	4.54±0.20***††	3.86±0.12	3.45±0.15
LVESD(mm)	3.50±0.22***††	2.79±0.08**	1.96±0.13
PWTd (mm)	0.60±0.03***	0.65±0.02**	0.76±0.01
PWTs (mm)	0.99±0.03***	1.03±0.03***	1.25±0.01
FS (%)	23.38±1.97***†	27.76±0.94***	43.43±1.38
r/h	3.80±0.24***††	3.00±0.18*	2.23±0.09

*P<0.01, **P<0.001, ***P<0.0001 vs Des+/+
†P<0.05; ††P<0.01 vs Des-/-
ANOVA analysis with Bonferrni post hoc test

Table S6. Echocardiography analysis of 4 months old mice

	**Des$^{-/-}$C3$^{-/-}$**	**Des$^{-/-}$**	**Wildtype**
	(n=12)	(n=17)	(n=12)
Heart Rate	554.00±13.19†	502.59±16.43	524.67±19.76
LVEDD(mm)	3.32±0.10††††	3.96±0.10***	3.18±0.07
LVESD(mm)	2.23±0.08**††††	2.78±0.10***	1.75±0.07
PWTd (mm)	0.67±0.01***†	0.70±0.01***	0.81±0.01
PWTs (mm)	1.06±0.02***	1.05±0.01***	1.24±0.01
FS (%)	32.90±0.55***	30.85±0.92***	45.23±1.43
r/h	2.49±0.06**††	2.82±0.09**	1.97±0.07

Values are mean±SE. LV, Left Ventricle, EDD, end-diastolic diameter; ESD, end-systolic diameter; FS, fractional shortening; PWT, posterior wall thickness at diastole (d) or systole (s); r/h, ratio of LV radius to PWT. n=number of animals analyzed.

*P<0.05, **P<0.001, ***P<0.0001 vs Des +/+

†P<0.05; ††P<0.01, †††P<0.001, ††††P<0.0001 vs Des-/-

ANOVA analysis with Bonferrni post hoc test

Table S7. Genetic profile of each ARVC patient in study.

ID	Gene	Transcript	Mutation
AC-1	NONE		
AC-2	NONE		
AC-3	TTN	NM_133378	p.W27187X
AC-4	NONE		
AC-5	DSG2	NM_001943	p.F531C
AC-6	NONE		
AC-7	TMEM43	NM_024334	p.S358L
AC-8	DSG2	NM_001943	p.Y198C
AC-9	DSG2	NM_001943	p.F531C
AC-10	DSG2	NM_001943	p.P142L
AC-11	Des	NM_001927	p.R127C
AC-12	LMNA	NM_005572	p.T266I
AC-13	NONE		
AC-14	NONE		
AC-15	DES	NM_001927	p.R406W
AC-16	TMEM43	NM_024334	p.X401R
AC-17	DSG2	NM_001943	p.F531C
AC-18	DSG2	NM_001943	p.F531C
AC-19	DSP	NM_004415	p.R1400X
AC-20	NONE		
AC-21	PKP2	NM_004572	p.R811fs; p.L703S
AC-22	PKP2	NM_004572	p.T851fs; p.S249T
AC-23	LMNA	NM_005572	p.L59M
AC-24	NONE		
AC-25	LMNA	NM_005572	p.R541H
AC-26	DSP,PKP2	DSP:NM_004415; PKP2:NM_004572	DSP:p.L1348R; PKP2:p.L394P
AC-27	PKP2	NM_004572	c.2300-2A>G
AC-28	PKP2	NM_004572	p.T785fs
AC-29	NONE		
AC-30	NONE		
AC-31	NONE		
AC-32	RYR2	NM_001035	p.I598V
AC-33	JUP	NM_002230	p.R142C
AC-34	DSG2	NM_001943	p.F531C

AC-35	PKP2	NM_004572	c.2146-1G>C:
AC-36	PKP2	NM_004572	p.K727X
AC-37	PKP2, TTN	PKP2:NM_004572; TTN:NM_133378	PKP2:p.P743fs; TTN:p.9299_9299del
AC-38	PKP2	NM_004572	p.S794A
AC-39	NONE		
AC-40	DSG2	NM_001943	p.P927L
AC-41	CTNNA3	NM_013266	p.G793E
AC-42	PKP2,TGFB3	PKP2:NM_004572; TGFB3:NM_003239	PKP2:p.Q49fs; TGFB3:p.E384K
AC-43	NONE		
AC-44	NONE		
AC-45	NONE		
AC-46	PKP2, CTNNA3	PKP2:NM_004572; CTNNA3:NM_013266	PKP2:p.R735X; CTNNA3:p.D602Y
AC-47	PKP2	NM_004572	p.R735X
AC-48	NONE		
AC-49	NONE		
AC-50	PKP2	NM_004572	p.S385fs
AC-51	TTN	NM_133378	p.E578fs
AC-52	PKP2	NM_004572	p.L431fs
AC-53	NONE		
AC-54	PKP2	NM_004572	c.1170+1G>A:
AC-55	NONE		
AC-56	NONE		
AC-57	NONE		
AC-58	PKP2, RYR2	PKP2:NM_004572; RYR2:NM_001035	PKP2:c.1170+1G>A; RYR2:p.R1154C;
AC-59	DSC2	NM_024422	p.L732fs
AC-60	PKP2	NM_004572	p.L738P
AC-61	DSG2	NM_001943	p.F531C
AC-62	NONE		
AC-63	NONE		
AC-64	PKP2, RYR2	PKP2:NM_004572; RYR2:NM_001035	PKP2:p.Y243X; RYR2:p.T2116A
AC-65	PKP2	NM_004572	c.2489+1G>A
AC-66	NONE		
AC-67	DSG2	NM_001943	p.F531C
AC-68	NONE		
AC-69	PKP2	NM_004572	c.2489+1G>A
AC-70	PKP2	NM_004572	p.I532fs
AC-71	NONE		

AC-72	PKP2	NM_004572	p.W290X
AC-73	DSC2	NM_024422	p.G77S
AC-74	PKP2	NM_004572	p.M716fs
AC-75	PNPLA2	NM_020376	p.G82D
AC-76	NONE		
AC-77	LMNA,DSG2	LMNA:NM_170707; DSG2:NM_001943	LMNA:p.Q308X; DSG2:p.L797Q
AC-78	TMEM43	NM_024334	p.X401R
AC-79	DSC2	NM_004949	p.G302D; p.R132H
AC-80	DSG2	NM_001943	p.R46W; p.Y198C
AC-81	NONE		
AC-82	DSP	NM_004415	p.R1838H
AC-83	DSG2	NM_001943	p.F102fs; p.P270R
AC-84	NONE		
AC-85	NONE		
AC-86	DSP	NM_004415	p.R1537H
AC-87	DSG2, CTNNA3	DSG2:NM_001943; CTNNA3:NM_013266	DSG2:p.R292C; CTNNA3:p.R484C

Table S8. Complement sC5b9 correlation with clinical characteristics in ARVC patients

Variables	Correlation (sC5b9)	P value
Weight (kg)	-0.081	0.486
Body mass index (kg/m2)	0.092	0410
Age (y)	0.152	0.177
Age at onset (y)	-0.008	0.945
AST (IU/L)	0.050	0.330
ALT (IU/L)	0.051	0.325
VE (/24h)	-0.240	0.054
MACE at baseline	-0.199	0.038
AF	0.335	0.007
LAAPD (mm)	0.337	0.001
IVS (mm)	0.044	0.346
LVEDD (mm)	-0.094	0.197

LVEF (%)	-0.233	0.016
RV ESV (ml)	0.272	0.035
RVEF (%)	-0.271	0.036
RVID (mm)	0.427	＜ 0.001
NYHA	0.339	0.002

ARVC = Arrhythmogenic right ventricular cardiomyopathy; VE = Ventricular extrasystole; MACE= major adverse cardiac events, including the syncope, implantable cardioverter-defibrillator therapy, sustained ventricular tachycardia; LAAPD = Left atrium anteroposterior diameter; IVS = Interventricular septal thickness; LVEDD = Left ventricular end diastolic diameter; LVEF = left ventricular ejection fraction; AF = Atrial fibrillation; RV ESV= Right ventricular end-systolic volume; RVEF = Right ventricular ejection fraction; RVID = Right ventricular internal dimension; NYHA = New York Heart Association.

Table S9. Baseline characteristics of ARVC patients with different concentration of sC5b9

**Characteristics**	**ARVC with sC5b9≤356 ng/ml (N=57）**	**ARVC with sC5b9>356 ng/ml (N=30)**	**P value**
Male (%)	39/57 (68.42%)	19/30 (63.33%)	0.641
Age (y)	39.54±1.61	42.69±3.20	0.328
Weight (kg)	66.76±1.90	68.28±2.35	0.628
Height(cm)	168.20±1.74	170.16±1.39	0.447
Body mass index (kg/m2)	23.81±0.83	23.36±0.78	0.733
Age at onset (y)	27.85±1.33	28.20±2.64	0.897
LAAPD (mm)	32.31±0.89	37.70±2.17	0.009
IVS (mm)	8.63±0.24	8.80±0.25	0.643
RVID (mm)	29.83±1.31	38.40±2.01	<0.001
LVEDD (mm)	47.27±1.08	52.10±1.52	0.013
LVEF (%)	55.74±1.77	48.39±3.07	0.028
Gene mutation (%)	36/57 (63.16%)	21/30 (70.00%)	0.637

Positive family history (%)	14/55 (25.45%)	5/30 (16.67%)	0.554
NYHA			0.075
I (%)	24/47 (51.06%)	9/30 (30.00%)	
II (%)	11/47 (23.40%)	7/30 (23.33%)	
III (%)	8/47 (17.02%)	5/30 (16.67%)	
IV (%)	4/47 (8.51%)	9/30 (30.00%)	
TWI (%)	10/46 (21.74%)	5/23 (21.74%)	0.999
AF (%)	5/57 (8.77%)	6/30 (20.00%)	0.134
NSVT (%)	45/51 (88.23%)	19/26 (73.08%)	0.093
ICD (%)	16/55 (29.09%)	11/30 (36.67%)	0.477

Data are expressed as either mean ± SEM or as number and percentage.

ARVC = arrhythmogenic right ventricular cardiomyopathy; LAAPD = Left atrium anteroposterior diameter; IVS = Interventricular septal thickness; RVID = Right ventricular internal dimension; LVEDD = Left ventricular end diastolic diameter; LVEF = left ventricular ejection fraction; NYHA=New York Heart Association; TWI= T wave inversion; AF = Atrial fibrillation; NSVT = None-sustained ventricular tachycardia; RFA = radiofrequency ablation;

Table S10. Cox regression analysis of all-cause mortality in ARVC patients.

Variables	Univariate Analysis		Multivariate Analysis	
	HR (95% CI)	p	HR (95% CI)	p
**Plasma sC5b9> 356 ng/ml**	**6.433(2.486~16.651)**	**<0.001**	**3.828(1.031~14.210)**	**0.045**
LVEDD, mm	1.020(0.970~1073)	0.442		
LVEF, %	0.924(0.857~0.996)	**0.038**	0.919(0.809~1.044)	0.192
RVID, mm	1.108(1.065~1.152)	**0.001**	1.094(0.940~1.273)	0.248
LAAPD, mm	1.079(0.953~1.222)	0.231		
NYHA	5.611(3.012~10.454)	**0.001**	**6.057(2.532~14.494)**	**0.001**
MACE at baseline	0.348(0.135~0.897)	**0.029**	1.159(0.060~22.364)	0.922
AF	7.568(2.982~19.206)	**<0.001**	2.357(0.185~30.028)	0.509

HR= hazard ratio, CI= confidence interval, ARVC = arrhythmogenic right ventricular cardiomyopathy; LVEDD = Left ventricular end diastolic diameter; LVEF = left ventricular ejection fraction; RVID = Right ventricular internal dimension; LAAPD = Left atrium anteroposterior diameter; NYHA=New York Heart Association; MACE= major adverse cardiac events, including the syncope, implantable cardioverter-defibrillator therapy, sustained ventricular tachycardia; AF = Atrial fibrillation.

Supplementary Figures:

Figure S1

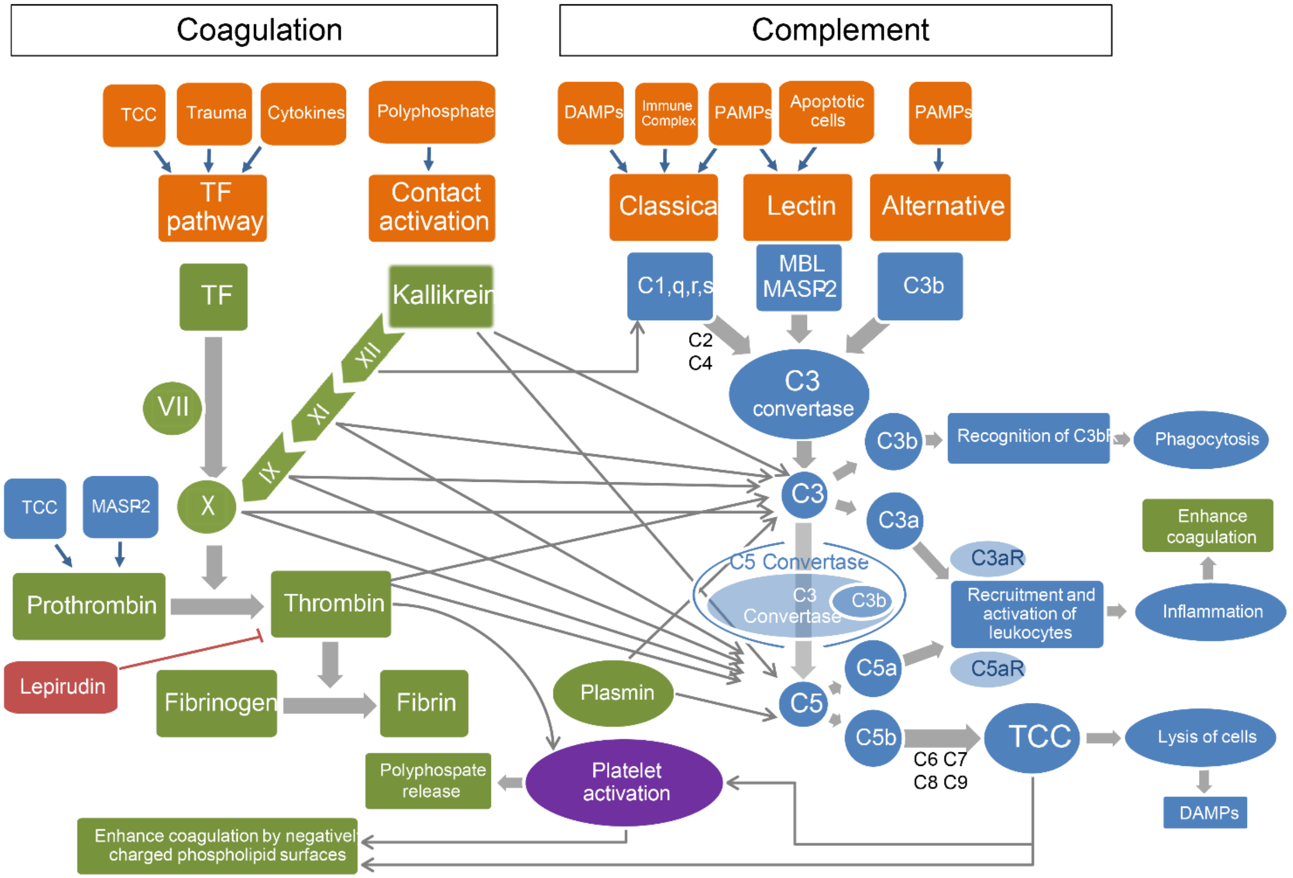

Figure S1. **Interplay between the coagulation/fibrinolysis cascades and the complement system.**

Both are composed of serine proteases that are activated through partial cleavage by an upstream enzyme. The coagulation cascade is divided into TF pathway and contact activation. The TF pathway could be activated by TCC, trauma, and some cytokines. Both pathways will merge at factor X level, which will generate thrombin. Thrombin could activate platelets and consequently induce platelet polyphosphate degranulation, thus consequently triggering contact activation. Activated platelets could additionally initiate the complement classical pathway by participating in C3 cleavage. Moreover, platelets contribute to the amplification of complement through the phosphorylation of C3b, which prolongs its life span. FXIIa can activate the classical complement pathway and kallikrein can activate both C3 and C5. Thrombin and plasmin can independently activate both C3 and C5. The final step of the coagulation process, catalyzed by thrombin, requires partial cleavage of soluble fibrinogen and polymerization to insoluble fibrin.

Complement is activated through the classical (DAMPs, immune complexes and PAMPs), lectin (PAMPs and apoptotic cells) or alternative (PAMPs) pathways all leading to C3 activation through C3 convertase which cleave C3 to C3a and C3b. C3b contributes to the formation of C5 convertase, which cleaves C5 to C5a and C5b. Subsequently, C5b will lead to TCC formation, which apart from cause lysis of microorganisms could also lyse host cells, releasing DAMPs. TCC will induce TF pathway and platelet activation, and will enhance coagulation by negatively charged phospholipid surfaces. C3a and C5a anaphylatoxins generated by complement activation will recruit and activate leukocytes, as well as induce platelet activation and aggregation, inducing thrombosis and inflammation, which are known to further enhance coagulation. Lepirudin: specific thrombin inhibitor. Blue: Components of complement system, Green: Components of coagulation/ fibrinolysis cascades, Orange: Initiatory components of pathways. Abbreviations: DAMPs, damage-associated molecular patterns; MASP-2, mannose-binding protein-associated serine protease 2; MBL, mannan-binding lectin; PAMPs, pathogen-associated molecular patterns; TCC, terminal complement complex; TF, tissue factor.

Sources used for Figure S2 generation[9-12]:

9. Markiewski MM, Nilsson B, Ekdahl KN, Mollnes TE, Lambris JD. Complement and coagulation: strangers or partners in crime? Trends Immunol. 2007; 28: 184-92.

10. Kurosawa S, Stearns-Kurosawa DJ. Complement, thrombotic microangiopathy and disseminated intravascular coagulation. J Intensive Care. 2014; 2: 65.
11. Amara U, Flierl MA, Rittirsch D, Klos A, Chen H, Acker B, et al. Molecular intercommunication between the complement and coagulation systems. J Immunol. 2010; 185: 5628-36.
12. Oikonomopoulou K, Ricklin D, Ward PA, Lambris JD. Interactions between coagulation and complement--their role in inflammation. Semin Immunopathol. 2012; 34: 151-65.

Figure S2

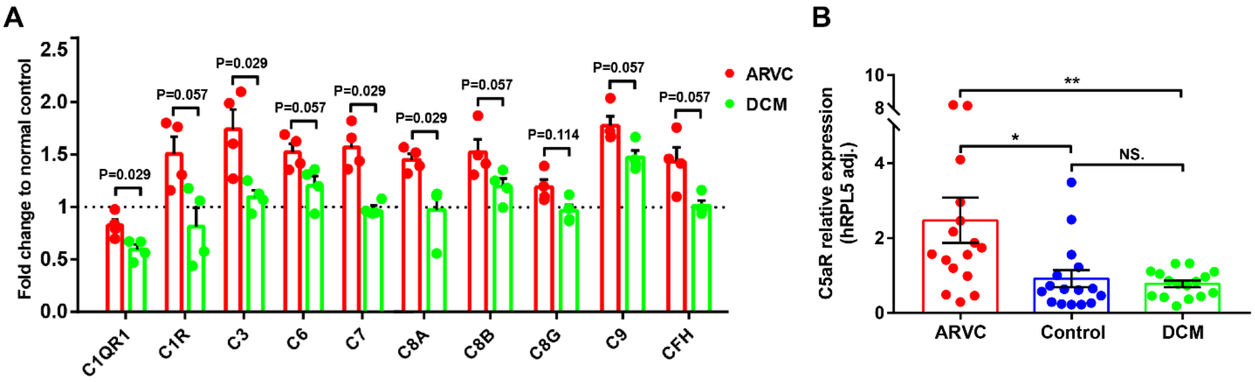

Figure S2. Elevated myocardial complement and coagulation factors in the right ventricle of ARVC hearts:

A. Proteome profiling demonstrated that complement system factors were significantly increased in ARVC patients right ventricular (RV) myocardium compared to non-diseased ventricle mixture (as the inner standards), (Mann-Whitney U test). **B.** Elevation of C5aR mRNA expression in cardiac tissue of ARVC patients compared to normal donors or DCM (n=16 separately; hRPL5 was used as the reference gene; P value adjusted by Tukeys multiple comparison test, Number of comparisons=3). * P<0.05; ** P<0.01, *** P<0.001, **** P<0.0001.

Figure S3

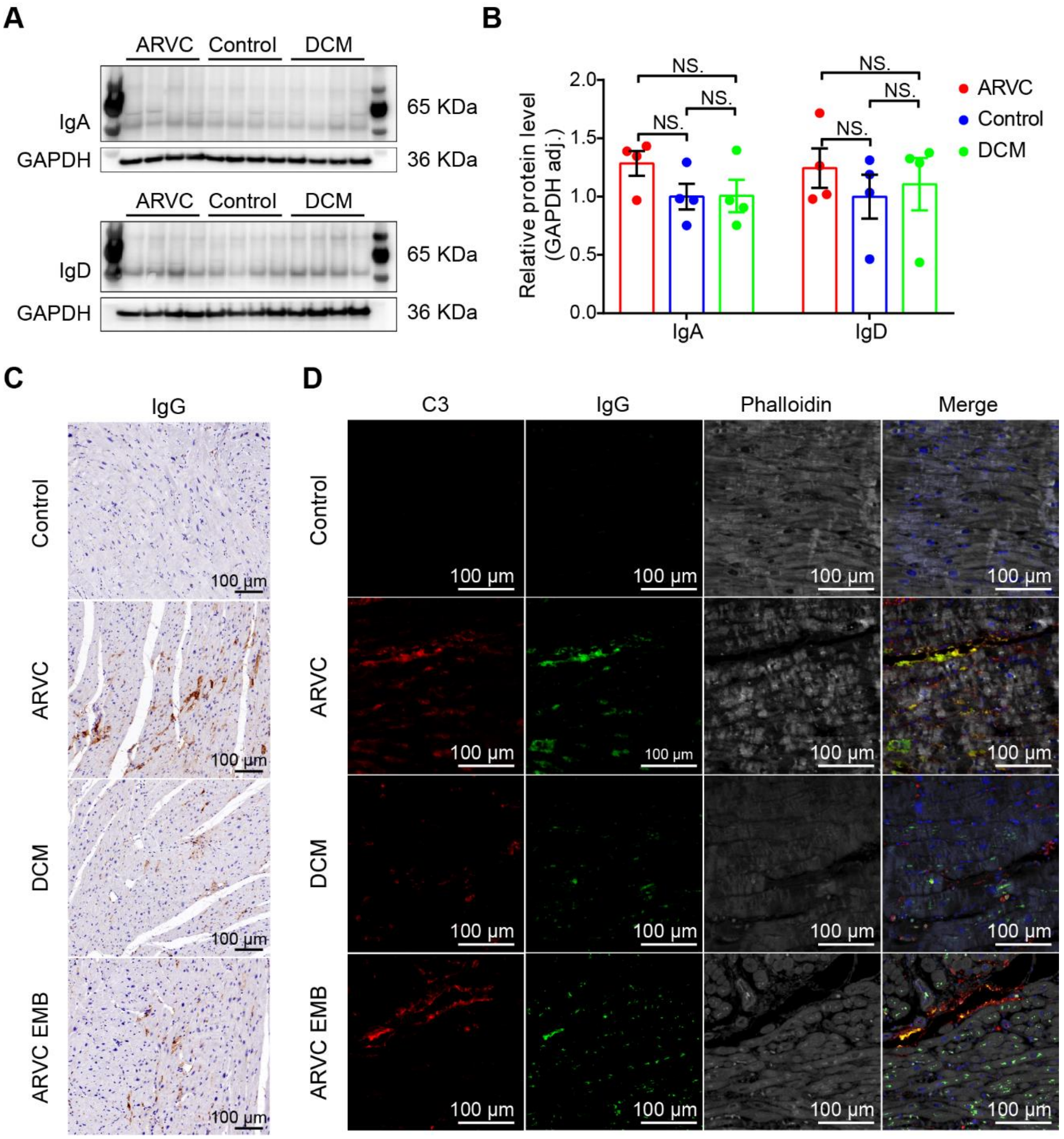

Figure S3. IgG co-deposited with complement factor in ARVC myocardium:

A, B. The expression of the IgA and IgD, was not significantly altered in ARVC patients in comparison to non-diseased or DCM hearts (P values adjusted by Tukeys multiple comparison correction, Number of comparisons=3). **C.** IHC analysis revealing increased deposition of IgG in ARVC myocardium. **D.** Immunofluorescence imaging showing the co-localization of IgG and C3 proteins in cardiomyocytes (cardiomyocytes were stained with phalloidin staining) in ARVC

(both the tissue of transplantation and endomyocardial biopsy -EMB-). * P<0.05; ** P<0.01, *** P<0.001, **** P<0.0001.

Figure S4

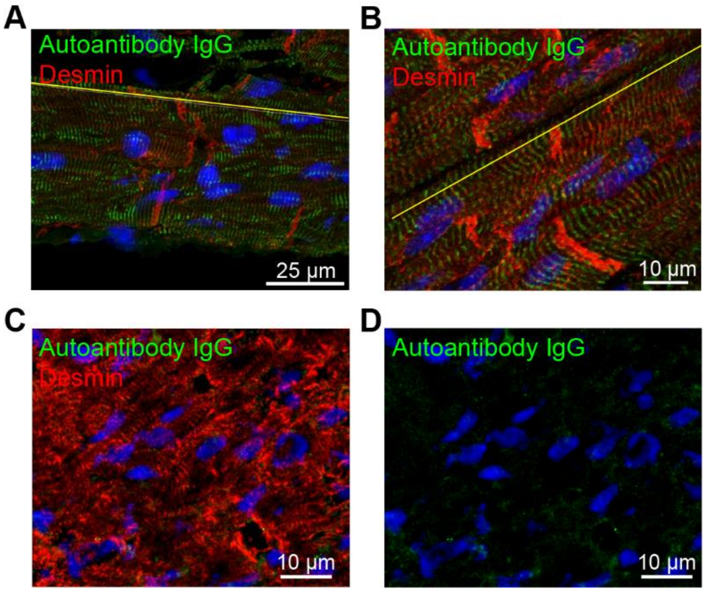

Figure S4. Serum from *Des*$^{-/-}$ mouse is positive for autoantibodies against z-line proteins. A, B: Cardiac tissue sections from WT animals stained with Des$^{-/-}$ serum IgG autoantibodies, (green, 1:100 dilution) reacting with z-line proteins. **C:** Cardiac tissue section from WT animal stained with serum IgG from *Des*$^{-/-}$ animal negative for autoantibodies (green, 1:100 dilution) **D**: same as **C**, but only green/blue channels shown. (Red staining: desmin; Blue: nuclei staining with DAPI).

Figure S5.

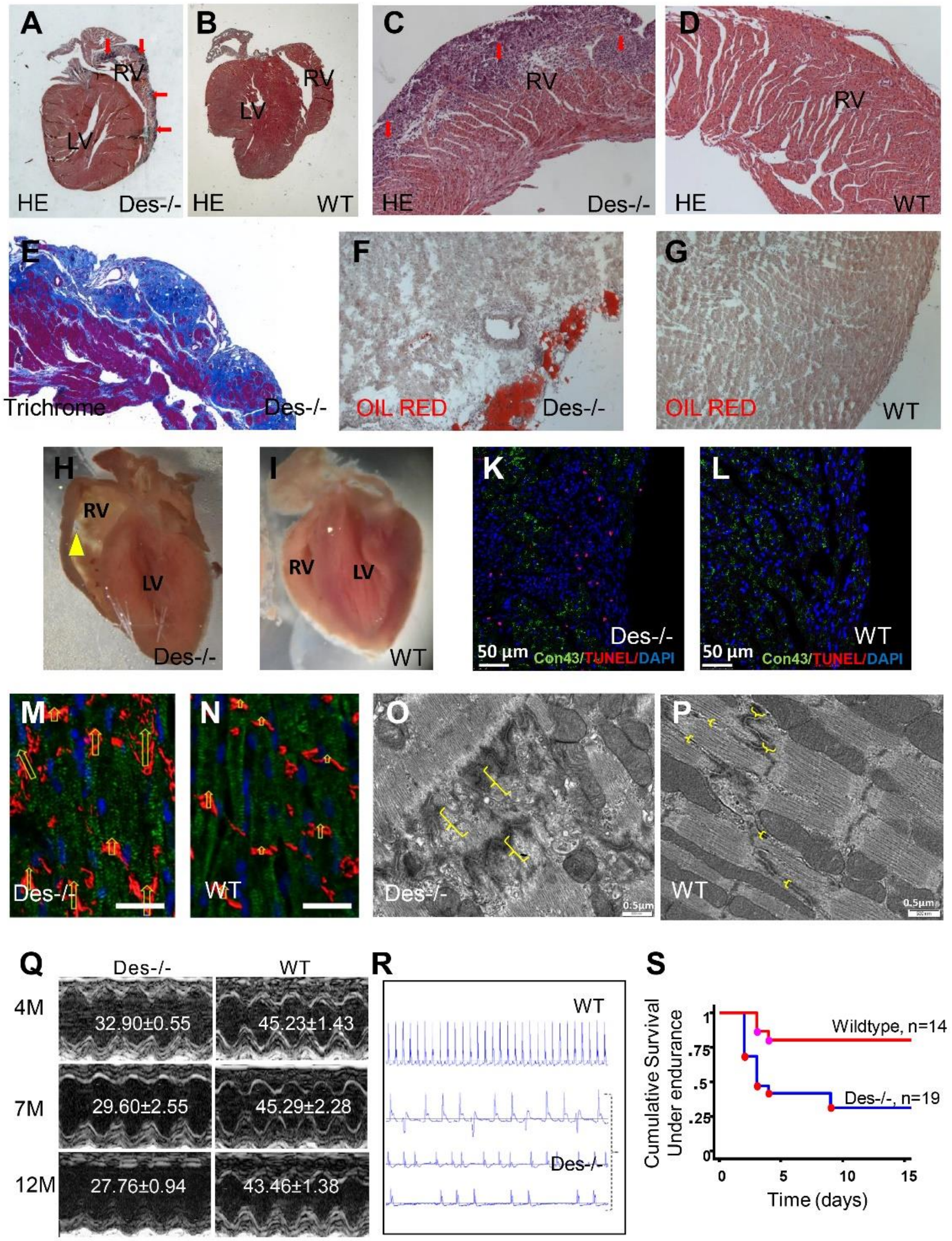

Figure S5. Clinicopathological findings indicating that Des-/- animals develop arrhythmogenic biventricular cardiomyopathy in consistence with the findings of currently available transgenic mice models of arrhythmogenic cardiomyopathy.

Cardiomyocytes degeneration is observed in the outer subepicardial myocardium of desmin-null mice which is extended towards the endocardium (**A** and **C**, red arrows), for comparison see WT, panels **B and D** (paraffin cardiac section, 24 days old animals, HE staining). This extended cardiomyocyte necrosis is followed by an inflammatory response and subsequently by injury repair and replacement of the necrotic fibers by dense collagenous scar (**E**, manson trichrome staining, 1.5 months old animals, collagen is blue).

Occasionally, limited positive staining for fatty cells (red staining) is also observed in areas of cardiac tissue injury and infiltration of inflammatory cells **F** (Oil red staining, 1.5 months old mice). **H**. As Des-/- animals age, dilation, aneurysm formation and rupture of the right ventricle (yellow arrow head) is occasionally observed, especially after endurance training (swimming) in the so called ‘‘triangle of dysplasia’’. **I.** wildtype heart, (8 months old animals). **K.** Increased apoptotic cell death (TUNEL positive nuclei) is observed in the myocardium of Des-/- animals **(**cardiac tissue sections from 24 days old animals, green staining is for connexin 43, red for TUNEL positive nuclei, and blue for nuclei –DAPI-). **L.** TUNEL staining of wildtype ones is negative. **M.** Abnormal intercalated disks are observed in Des-/- cardiomyocytes, as they appear thicker and more convoluted (immunofluorescence staining of frozen cardiac tissue sections, red: N cadherin z-stack projection, green: sarcomeres marker, blue: DAPI) and (**O)** electron microscopy of Des-/- cardiomyocytes. The corresponding analysis of wild type animals are indicated at (**N)** and (**P). Q.** Progressive development of heart failure was evident in the desmin-null animals as they age. M-mode echocardiography indicated major decrease of left ventricle fractional shortening (FS, %). **R.** Electrocardiographic recordings displayed a major increase in premature ventricular beats in the Des-/- mice. Supraventricular ectopic beats were also apparent. Furthermore, atrioventricular block is observed in the Des-/- mice during endurance training, in addition to premature ventricular beats after which, usually the Des-/- mice died. **S.** Reduced survival rate is observed in Des-/- mice compared to wildtype ones at the end of a 15-days swimming stress testing (log rank $\chi 2$ 6.35, p=0.01).

Figure S6

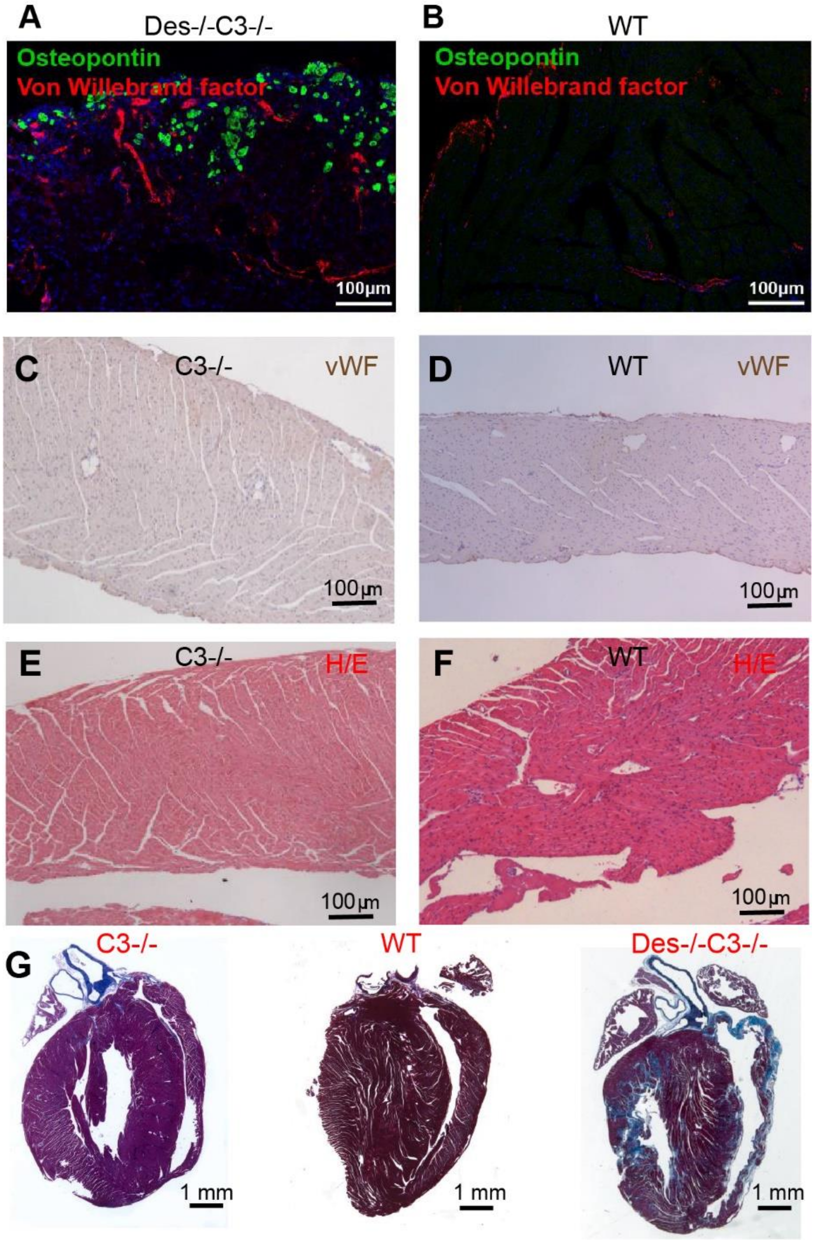

Figure S6. A. Extensive von Willebrand factor (red) staining is observed in cardiac tissue sections from Des-/-C3-/- animals in areas with cardiomyocyte degeneration, infiltration of inflammatory cells, and dystrophic calcification, whereas in wildtype (WT) **(B)** there is minimum staining. (Green staining is for osteopontin, a marker of dystrophic calcification). **C.** Von Willebrand factor staining in C3-/- animals is minimum as in WT **(D). E**. Hematoxylin eosin staining of C3-/- cardiac tissue sections indicate a similar tissue morphology to WT **(F). G.**

Whole hearts sections staining with Manson trichrome indicates a similar pattern for C3-/- and wildtype animals, in contrast to Des-/-C3-/- where increased replacement fibrosis (blue) and RV dilation is observed.

Figure S7

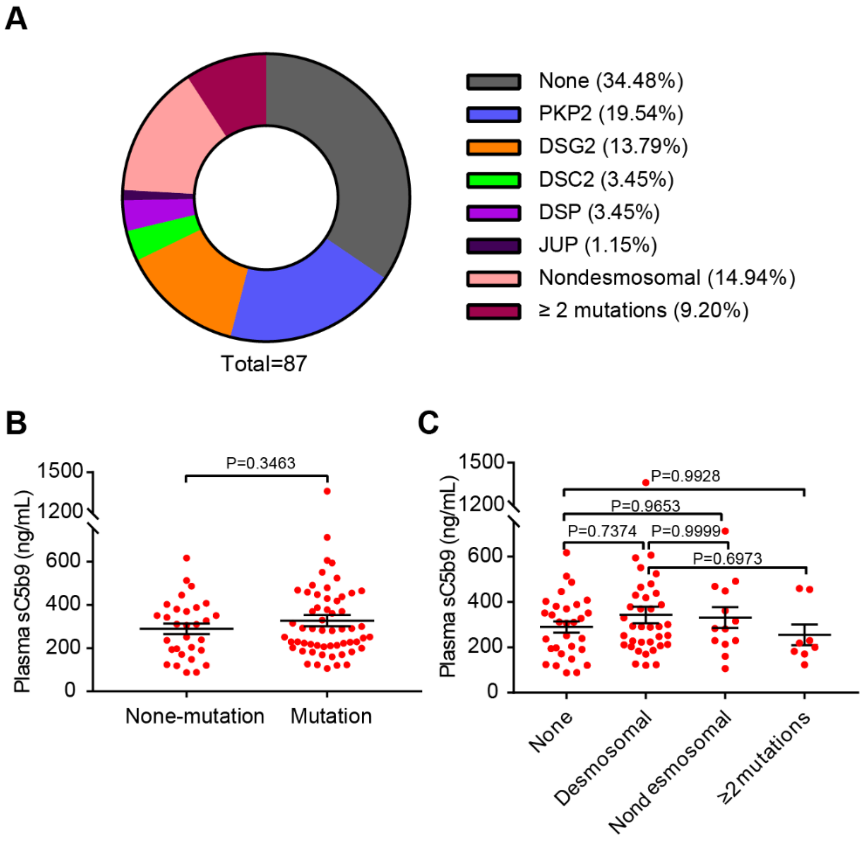

Figure S7. The plasma sC5b9 levels with genetic mutation in ARVC patients

A. Genetic mutation status of ARVC patients (n = 87). **B.** There is no significant difference in the plasma C5b9 levels between ARVC patients with or without gene mutations. (Student's t-test). **C.** The plasma sC5b9 level**s** had no significant association with the underlying genetic mutation in ARVC patients (P value adjusted by Tukeys multiple comparison test, Number of comparisons=5). * P<0.05; ** P<0.01, *** P<0.001, **** P<0.0001.

Figure S8

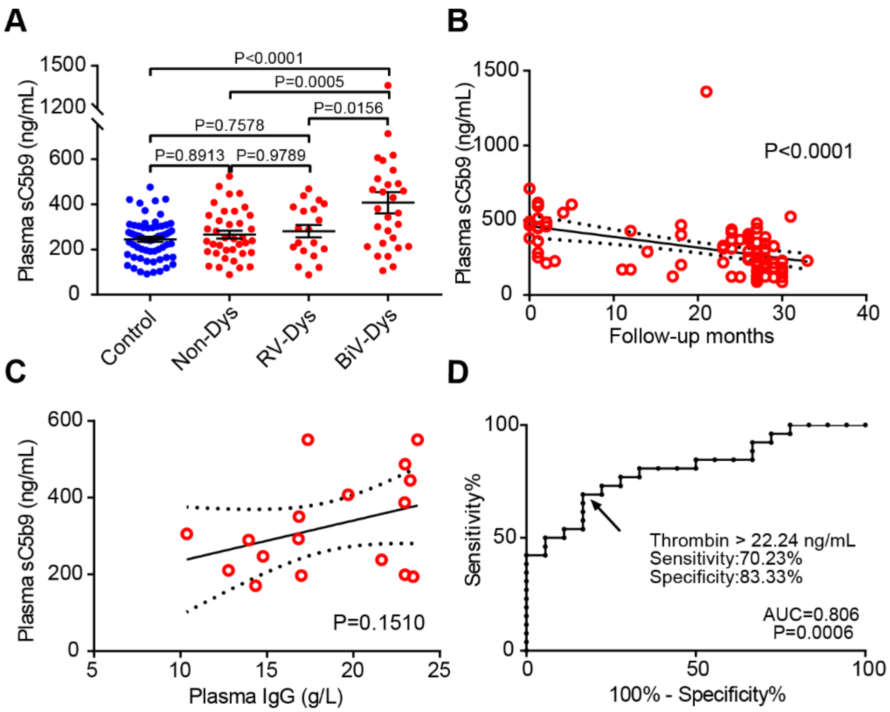

figure S8. Circulating complement and coagulation factor are elevated in ARVC patients. **A.** sC5b9 was significantly up-regulated in ARVC patients with bi-ventricular dysfunction. (P value adjusted by Sidak's multiple comparison test, Number of comparisons=5). **B.** Elevated plasma sC5b9 levels were negatively correlated to poor prognosis in ARVC patients. **C.** A positive correlation is observed between the plasma sC5b9 and IgG levels in ARVC patients. **D.** ROC curve to distinguish ARVC patients and healthy volunteer according to the plasma thrombin level. * P<0.05; ** P<0.01, *** P<0.001, **** P<0.0001.
